# Mechano-signaling via Piezo1 prevents activation and p53-mediated senescence of muscle stem cells

**DOI:** 10.1016/j.redox.2022.102309

**Published:** 2022-04-02

**Authors:** Yundong Peng, Jingjing Du, Stefan Günther, Xinyue Guo, Shengpeng Wang, Andre Schneider, Li Zhu, Thomas Braun

**Affiliations:** a Max-Planck-Institute for Heart and Lung Research, Department of Cardiac Development and Remodeling, 61231, Bad Nauheim, Germany; b College of Animal Science and Technology, Sichuan Agricultural University, Chengdu, 611130, China; c Cardiovascular Research Center, School of Basic Medical Sciences, Xi'an Jiaotong University Health Science Center, Key Laboratory of Environment and Genes Related to Diseases, No.76 West Yanta Road, Yanta District, Xi'an, China; d German Centre for Cardiovascular Research (DZHK), Partner Site Rhein-Main, Frankfurt am Main, Germany

**Keywords:** Muscle stem cells, Mechanosensing, Senescence, p53, Skeletal muscle regeneration

## Abstract

Skeletal muscle stem cells (MuSCs), also called satellite cells, are instrumental for postnatal muscle growth and skeletal muscle regeneration. Numerous signaling cascades regulate the fate of MuSCs during muscle regeneration but the molecular mechanism by which MuSCs sense mechanical stimuli remain unclear. Here, we describe that Piezo1, a mechanosensitive ion channel, keeps MuSCs in a quiescent state and prevents senescence. Absence of Piezo1 induces precocious activation of MuSCs, attenuates proliferation, and impairs differentiation, essentially abolishing efficient skeletal muscle regeneration and replenishment of the MuSC pool. Furthermore, we discovered that inactivation of *Piezo1* results in compensatory up-regulation of T-type voltage-gated Ca2+ channels, leading to increased Ca^2+^ influx, which strongly induces NOX4 expression via cPKC. Elevated NOX4 expression in *Piezo1*-deficient MuSCs increases ROS levels and DNA damage, causing P53-dependent cellular senescence and cell death. The importance of the P53/P21-axis for mediating Piezo1-dependent cellular defects was confirmed by pharmacological inhibition of P53 in *Piezo1*-deficient mice, which abrogates increased senescence of muscle cells and normalizes muscle regeneration. Our findings uncover an essential role of Piezo1-mediated mechano-signaling in MuSCs for maintaining quiescence and preventing senescence. Reduced mechano-signaling due to decreased physical activity during aging may contribute to the increase of senescent cells and the decline of MuSC numbers in geriatric mice and humans.

## Highlights

• Piezo1 is highly expressed in skeletal MuSCs and prevents their precocious activation.
• Loss of Piezo1 increases Ca^2+^ influx into MuSCs, which induces NOX4 expression via PKC, leading to enhanced ROS generation.
• Inactivation of Piezo1 depletes the MuSC pool and causes P53-dependent senescence of MuSCs.
• ROS scavenging in Piezo1-deficient MuSCs prevents P53 accumulation.
• Inhibition of P53 mitigates skeletal muscle regeneration defects in mice with Piezo1-deficient MuSCs.

## Graphical abstract

**Figure:**
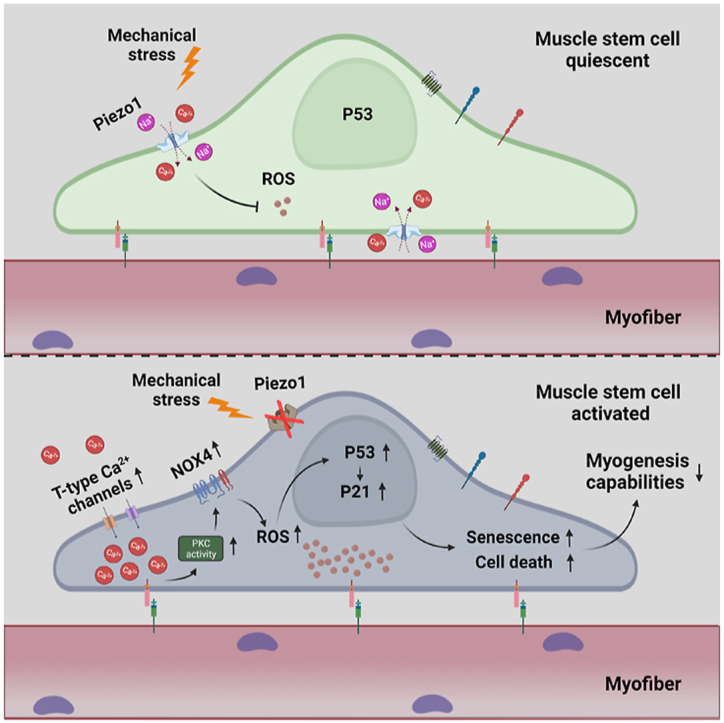

## 1 Introduction

Skeletal muscle regeneration requires MuSCs, a population of adult stem cells capable of self-renewal and myogenic differentiation. MuSCs are mostly quiescence in adult skeletal muscles under homeostatic conditions and reside between the sarcolemma and basal lamina of myofibers [1]. Quiescent MuSCs in skeletal muscles are marked by expression of PAX7 and undergo activation in response to injury or other stimuli such as intense physical exercise [[2], [3], [4], [5]]. MuSCs rapid upregulate myogenic transcription factors (e.g. MYF5, MYOD) during activation and differentiate following proliferation by fusing to each other or to existing myofibers [6]. A subpopulation of activated MuSCs escapes differentiation and returns to quiescence, allowing repopulation of the muscle stem cell niche [[7], [8], [9]]. Major efforts have been made to understand the mechanism determining MuSC quiescence and activation. Numerous signal pathways were identified that play critical roles in this process, including Notch, mTOR, and P38/MAPK [[9], [10], [11], [12]]. However, it is still not fully understood how MuSCs sense dynamic changes in the microenvironment and how the decision is made whether to stay in a quiescent state or to initiate activation. Changes within the MuSC niche are considered to affect MuSCs and to direct fate decisions.

Damaged, unwanted, or superfluous MuSCs may submit to programmed cells death or undergo senescence [13]. Cellular senescence is characterized by permanent cessation of cell division, accompanied by distinct phenotypic changes including metabolic dysfunction, chromatin remodeling, increased autophagy, and a pro-inflammatory senescence-associated secretory phenotype (SASP) [14,15]. Acquisition of senescence plays a physiological role during normal development, but enhanced accumulation of senescent cells contributes to age-related pathological processes [[16], [17], [18]]. Cellular senescence can be initiated by numerous processes including telomere erosion, DNA damage, mitochondrial dysfunction, and accumulation of oxygen reactive species (ROS) [15,19,20]. Senescence-induced cell cycle arrest is driven by two tumor suppressor pathways, P16/RB and P53/P21. Although increased presence of P16 is usually considered as a hallmark of senescence, some cell types are more responsive to P53/P21 compared to P16/RB for initiation of senescence, depending on the cellular context [19,21]. During aging, MuSCs undergo enhanced rates of cellular senescence, which has been associated with lowered muscle performance and decreased muscle regeneration [22,23] Accumulation of senescent MuSCs is promoted by age-related structural and molecular changes of the MuSC niche, which impede functions of MuSCs [24]. Reduced exercise-dependent mechanical load during the course of aging may contribute to changes of the MuSC niche. However, it is currently not known whether and how MuSCs directly sense exercise-induced mechanical load and whether constant sensing of mechanical signals is indispensable for MuSCs to avoid cellular senescence.

Piezo1 is mechanosensitive cation channel, which opens when membrane tension increases [25], a process that is instrumental for responding to mechanical load in various cell types [[26], [27], [28], [29]]. Numerous *in vivo* studies demonstrated the critical mechanosensitive function of Piezo1 in various physiological processes, including somatosensation, hearing, bone formation, adipogenesis, vascular development, and the control of blood pressure and heart functions, [25,[28], [29], [30], [31], [32], [33]]. Piezo1 is considered to have irreplaceable roles in tissue homeostasis but relatively little is known about its function in skeletal muscles. Previous *in vitro* studies, using primary myoblasts and the C2C12 myoblast cell line, unraveled a function of Piezo1 in muscle cell differentiation, while *in vivo* studies with a focus on MuSCs are lacking so far [[34], [35], [36]].

Here, we demonstrate a critical role of Piezo1 for reducing ROS formation and preventing P53-dependent senescence of MuSC. We show that Piezo1 is required to keep MuSCs in a quiescent state but also for myoblast proliferation and differentiation, which makes Piezo1 indispensable for normal skeletal muscle regeneration. In a more general sense, the study uncovers how mechanical signal transduction reduces cellular senescence in a population of somatic stem cells and emphasizes the importance of mechanical cues for skeletal muscle physiology.

## 2 Results

### 2.1 *Piezo1* is preferentially expressed in quiescent MuSC

**Fig. 1:**
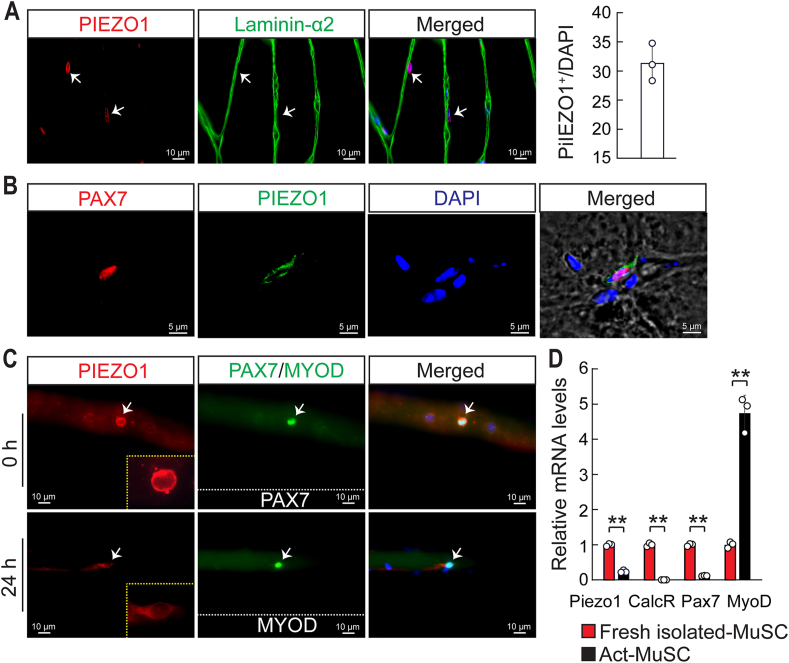
**Piezo1 is strongly expressed in quiescent MuSC. (A)** Immunofluorescence analysis of Piezo1 (red) laminin-2 (green) and DAPI (blue) on sections of WT TA muscles. Quantifications are shown on the right (n = 3, 2-months-old male mice). **(B)** Immunofluorescent analysis of Piezo1 (red) PAX7 (green) and DAPI (blue) on sections of WT TA muscles. **(C)** Immunofluorescence analysis of Piezo1 (red) PAX7/MYOD (green) and DAPI (blue) on isolated myofibers from WT FDB muscles at 0h or 24h of culturing. **(D)** RT-qPCR analysis of *Piezo1* expression in freshly isolated MuSCs (Fresh isolated-MuSC) and 24h after culturing (Act-MuSC). Expression levels were normalized to *Gapdh* (*t*-test: **p < 0.01, n = 3, 2-months-old male mice). (For interpretation of the references to color in this figure legend, the reader is referred to the Web version of this article.)

To analyze whether MuSCs may sense mechanical load via Piezo1, we performed immunofluorescence staining for Piezo1 on sections of tibialis anterior (TA) muscles (Fig. 1A). Co-staining for Piezo1 and PAX7, a MuSC marker, revealed that most PAX7^+^ cells express Piezo1 while the sarcolemma of myofibers did not show obvious Piezo1 staining, indicating that expression of Piezo1 within the muscle lineage is confined to MuSCs (Fig. 1A and B). To confirm this finding, we analyzed freshly isolated single myofibers. Piezo1 expression was detected in PAX7^+^ cells immediately (0h) after myofiber isolation (Fig. 1C) and to a lower degree in activated, MYOD-expressing MuSCs after 24h of cultivation (Fig. 1C). Next, we determined mRNA levels of *Piezo1* by RT-qPCR in freshly isolated MuSC, purified by fluorescence activated cell sorting (FACS), and in MuSCs after cultivation for 24h. Piezo1 was highly expressed in freshly isolated MuSCs but expression declined substantially during the culture period, concomitant with increased expression of the MuSC activation marker MYOD (Fig. 1D). Taken together the results indicate that *Piezo1* is specifically expressed in quiescent MuSCs and declines during activation and differentiation.

### 2.2 *Piezo1* is required for maintaining quiescence of MuSCs and to avoid depletion of the MuSC pool

The decline of *Piezo1* expression in activated compared to quiescent MuSCs suggested that *Piezo1* might secure MuSC quiescence, which is required for long-term maintenance and self-renewal of the MuSC pool, allowing efficient muscle regeneration at later stages of life. To analyze this possibility, we treated explanted myofibers carrying attached MuSCs and freshly isolated MuSCs with Yoda1, a specific activator of Piezo1. Treatment with Yoda1 attenuated activation of MuSCs as indicated by reduced ratios of PAX7^+^MYOD^+^/PAX7^+^ cells in both cultured myofibers and MuSCs (Fig. S1), suggesting that activation of Piezo1 maintains quiescence of MuSCs.

To further test this hypothesis, we inactivated *Piezo1* in PAX7^+^ cells *in vivo* by breeding *Pax7*-Cre (*Pax7*^*ICN*^) mice with a strain in which the *Piezo1* gene is flanked by loxP sites (*Piezo1*^*f/f*^) to generate *Pax*^*ICN*^/*Piezo1*^*f/f*^ mice (hereafter named *Piezo1*^*mKO*^). RT-qPCR analysis of MuSCs isolated from *Piezo1*^*mKO*^ mice confirmed efficient inactivation of *Piezo1*, reducing expression of *Pizeo1* by approximately 94% in FACS-purified, freshly isolated *Piezo1*^*mKO*^ MuSCs compared to control *Piezo1*^*f/f*^ or *Pax7*^*ICN*^ MuSCs (Fig. S2A). Importantly, FACS analysis of hind limb muscles revealed a marked reduction of the number of *Piezo1*^*mKO*^ MuSCs compared to control hind limb muscles (Fig. S2B). Consistent with this observation, we detected an approximately 80% reduction of the numbers of PAX7^+^ MuSCs in TA muscles of *Piezo1*^*mKO*^ (Fig. S2C) suggesting that failure of mechanosensing due to inactivation of *Piezo1* during muscle development depletes the MuSC pool, probably due to precocious activation of MuSCs or the failure to acquire a quiescent state. Only 60% of the remaining PAX7^+^ MuSCs in *Piezo1*^*mKO*^ TA muscles expressed CALCR, a marker for MuSC quiescence, while virtually all PAX7^+^ MuSCs showed CALCR expression under sedentary conditions in control muscles (Fig. S2D). Moreover, approximately 40% of the remaining MuSCs in *Piezo1*^*mKO*^ TA muscles expressed MYOD, confirming that *Piezo1* plays a critical role for the regulation of MuSC quiescence (Fig. S2E). Of note, control experiments ascertained that mouse strains carrying *Piezo1*^*f/f*^ alleles or expressing Cre-recombinase in MuSCs do neither show a reduction of PAX7^+^ MuSCs (Figs. S3A and B) nor changes in skeletal muscle morphology (Figs. S3C–E).

**Fig. 2:**
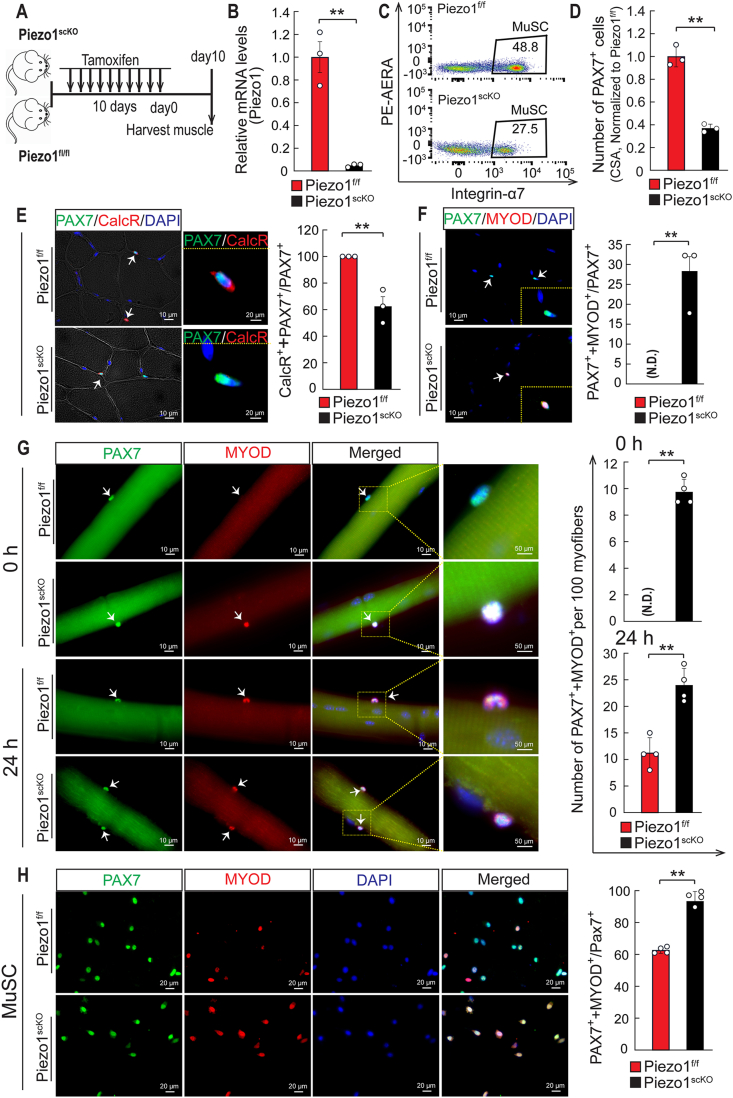
***Piezo1* prevents activation and depletion of MuSC. (A)** Schematic representation of the experimental design. **(B)** RT-qPCR analysis of *Piezo1* expression in MuSCs after tamoxifen treatment. mRNA levels were normalized to *Gapdh* (*t*-test: **p < 0.01, n = 3, 2-months-old male mice). **(C)** FACS analysis of MuSCs in *Piezo1*^*f/f*^ (upper) and *Piezo1*^*scKO*^ (lower) muscles. The percentage of MuSCs is shown in the panels (n = 3, 2-months-old male mice). **(D)** Quantification of PAX7^+^ MuSCs on TA muscle sections from *Piezo1*^*f/f*^ and *Piezo1*^*scKO*^ mice (*t*-test: **p < 0.01, n = 3, 2-months-old male mice, 3 sections per sample). **(E)** Immunofluorescence staining for CALCR (red) and PAX7 (green) on TA muscle sections from *Piezo1*^*f/f*^ and *Piezo1*^*scKO*^ mice. DAPI stains nuclei (blue). Quantifications are shown on the right (*t*-test: **p < 0.01, n = 3, 2-months-old male mice, 3 sections per sample). **(F)** Immunofluorescence staining for MYOD (red) and PAX7 (green) on TA muscle sections from *Piezo1*^*f/f*^ and *Piezo1*^*scKO*^ mice. DAPI stains nuclei (blue) (*t*-test: **p < 0.01, N.D., not detected, n = 3, 2-months-old male mice, 3 sections per sample). **(G)** Immunofluorescent analysis of MYOD (red), PAX7 (green) and DAPI (blue) on isolated myofibers from FDB muscles of *Piezo1*^*f/f*^ and *Piezo1*^*scKO*^ mice at 0h (upper panel) and 24h (lower panel) of culturing. Quantifications are shown on the right (*t*-test: **p < 0.01, N.D., not detected, n = 4, 2-months-old male mice, ≥100 randomly chosen myofibers per sample). **(H)** Immunofluorescence staining of MuSCs for MYOD (red), PAX7 (green), and DAPI (blue) after 5 days of culture. Quantifications are shown on the right (*t*-test: **p < 0.01, n = 4, 2-months-old male mice, 20 fields per sample). (For interpretation of the references to color in this figure legend, the reader is referred to the Web version of this article.)

To exclude developmental defects caused by deletion of *Piezo1*, we also inactivated *Piezo1* in adult MuSCs using a tamoxifen (TAM)-inducible *Pax7*-Cre allele (*Pax7*^*CreERT2*^). Efficient inactivation of *Piezo1* was accomplished by 10 daily injections of TAM into *Pax7*^*CreERT2*^/*Piezo1*^*f/f*^ mice (hereafter named *Piezo1*^*scKO*^), which lowered *Piezo1* expression to nearly undetectable levels compared to control mice (*Piezo1*^*f/f*^) (Fig. 2A and B). FACS analysis unveiled a reduction of MuSCs by approximately 50% in *Piezo1*^*scKO*^ compared to control mice, which corresponded to a 60% reduction of PAX7^+^ MuSCs on sections of TA muscles (Fig. 2C and D). In addition, we observed increased activation of the remaining MuSCs in *Piezo1*^*scKO*^ muscles as indicated by a 40% reduction of CALCR^+^ PAX7^+^ relative to all PAX7^+^ cells while the number of MYOD-expressing PAX7^+^ MuSCs in *Piezo1*^*scKO*^ muscles increased to approximately 25% (Fig. 2E and F). Along the same line, analysis of cultured single myofibers immediately after plating (0h) identified MYOD^+^ PAX7^+^ cells on fibers from *Piezo1*^*scKO*^ but not from *Piezo1*^*f/f*^ control mice (Fig. 2G). Although MYOD^+^ PAX7^+^ cells emerged on fibers from control mice after cultivation for 24h, the number was substantially higher on fibers from *Piezo1*^*scKO*^ mice (Fig. 2G). MYOD^+^ PAX7^+^ cells were also more abundant in MuSCs isolated from *Piezo1*^*scKO*^ compared to *Piezo1*^*f/f*^ mice, albeit the difference was less pronounced than in the myofiber cultures (Fig. 2H). Taken together, these results indicate that *Piezo1* is instrumental for keeping MuSCs in quiescence and for maintaining the MuSC pool in skeletal muscles.

### 2.3 *Piezo1* supports proliferation and differentiation of isolated MuSCs *in vitro*

**Fig. 3:**
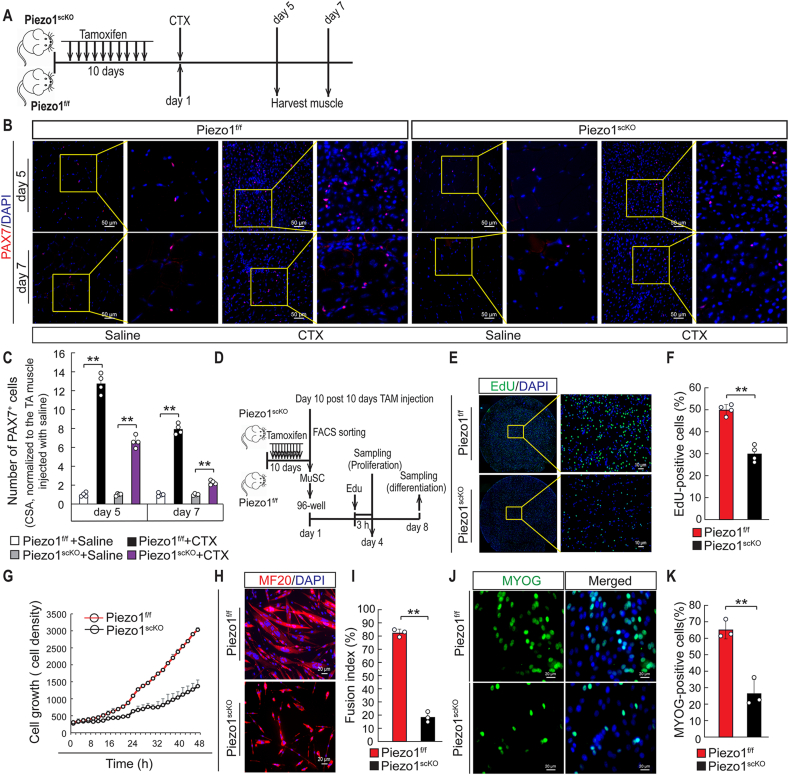
**Inactivation of *Piezo1* impairs proliferation and differentiation of MuSC. (A)** Schematic outline of the experimental design. **(B, C)** Immunofluorescence staining for PAX7^+^ MuSCs on TA muscle sections from *Piezo1*^*f/f*^ and *Piezo1*^*scKO*^ mice. The number of MuSCs was determined 5 and 7days after CTX treatment (B) and (C). Quantifications are shown in the lower panel. For each whole cross section aera (CSA), all PAX7^+^ MuSCs were counted. The number of PAX7^+^ MuSCs in CTX injected TA muscle were normalized to saline-injected TA muscles of *Piezo1*^*f/f*^ or *Piezo1*^*scKO*^ mice. (One-way ANOVA: **p < 0.01, n = 4, 2-months-old male mice, 3 sections per sample). **(D)** Schematic outline of the experimental design. **(E)** Detection of EdU^+^ in PAX7^+^ MuSCs from *Piezo1*^*f/f*^ and *Piezo1*^*scKO*^ mice after day 4 of culture. **(F)** Quantification of EdU^+^ MuSCs is shown on the right (*t*-test: **p < 0.01, n = 4, 2-months-old male mice, 4 samples per mouse). **(G)** Time lapse imaging of MuSCs proliferation. MuSCs from *Piezo1*^*f/f*^ are represented by the red line, MuSCs from *Piezo1*^*scKO*^ by the black line. **(H, I)** Immunofluorescence staining for myosin heavy chain (MF20 antibody, green) and DAPI (blue) to determine the fusion index of *Piezo1*^*f/f*^ and *Piezo1*^*scKO*^ MuSCs after 8 days of culture (H) and quantification (I) (*t*-test: **p < 0.01, n = 3, 2-months-old male mice, 3 samples per mouse, 10 randomly chosen fields per sample). **(J, K)** Immunofluorescence staining for MYOG (green) and DAPI (blue) of *Piezo1*^*f/f*^ and *Piezo1*^*scKO*^ MuSCs after 8 days of culture (J) and quantification (K) (*t*-test: **p < 0.01, n = 3, 2-months-old male mice, 4 samples per mouse). (For interpretation of the references to color in this figure legend, the reader is referred to the Web version of this article.)

Although expression of *Piezo1* declines in activated and proliferating MuSCs, we wanted to know whether *Piezo1* does also play a role for proliferation and differentiation of MuSC, either due to precocious activation of MuSCs or due to a function during proliferation. *Piezo1* was inactivated in adult MuSCs by 10-times injection of TAM, which was verified by immunofluorescence staining of isolated PAX7^+^ MuSCs with a Piezo1 antibody. We detected a substantial decrease of PAX7^+^ MuSCs in regenerating TA muscles of *Piezo1*^*scKO*^ compared to *Piezo1*^*f/f*^ control mice, 5 and 7 days following cardiotoxin (CTX) injection to induce muscle injury (Fig. 3A–C). Similar results were obtained when assessing the proliferation rate of MuSCs *in vitro*. MuSCs isolated from the hind limbs of adult control and TAM-induced *Piezo1*^*scKO*^ mice were cultured in growth medium for 4 days and then pulsed with the thymidine analog EdU for 3 h (Fig. 3D). EdU incorporation in *Piezo1*^*scKO*^ MuSCs was reduced by approximately 50% compared to *Piezo1*^*f/f*^ control MuSCs (Fig. 3E and F), which corresponded to a reduced proliferation rate monitored by time-lapse imaging (Fig. 3G). We also observed a substantially decline of the fusion index and percentage of MYOG positive nuclei in *Piezo1*^*scKO*^ MuSCs after placement into differentiation medium, indicating compromised differentiation (Fig. 3H–K).

Since reduction of PAX7^+^ MuSCs in regenerating TA muscles of *Piezo1*^*scKO*^ and attenuated proliferation of *Piezo1*^*scKO*^ MuSCs may be caused by precocious activation of MuSCs *in vivo*, we turned to a different experimental system. We isolated MuSCs by FACS from adult *Piezo1*^*f/f*^ muscles and then treated cultured MuSCs with Adenovirus-CRE (Ad-Cre) to induce inactivation of *Piezo1 in vitro* (Fig. S4A). RT-qPCR analysis revealed successful inactivation of *Piezo1* in cultured MuSCs after 48h exposure to Ad-Cre but not Ad-Null (Fig. S4B). Inactivation of *Piezo1 in vitro* essentially yielded the same results as observed *in vivo* and when culturing isolated MuSCs that had lost *Piezo1* expression *in vivo*. We found a substantial reduction of EdU incorporation and a severe reduction of the proliferation rate in adult *Piezo1*^*f/f*^ MuSCs treated with Ad-Cre compared to the Ad-Null group (Figs. S4C and D). Furthermore, MuSCs in which *Piezo1* was inactivated *in vitro* during the proliferation phase formed fewer multinucleated myotubes, also showing decreased length and reduced percentage of MYOG positive nuclei (Figs. S4E–I). These results clearly indicate that *Piezo1* does not only suppress activation of MuSCs but also plays an important role at later stages to enable proliferation and differentiation of MuSC.

### 2.4 Expression of *Piezo1* in MuSCs is necessary for efficient skeletal muscle regeneration

**Fig. 4:**
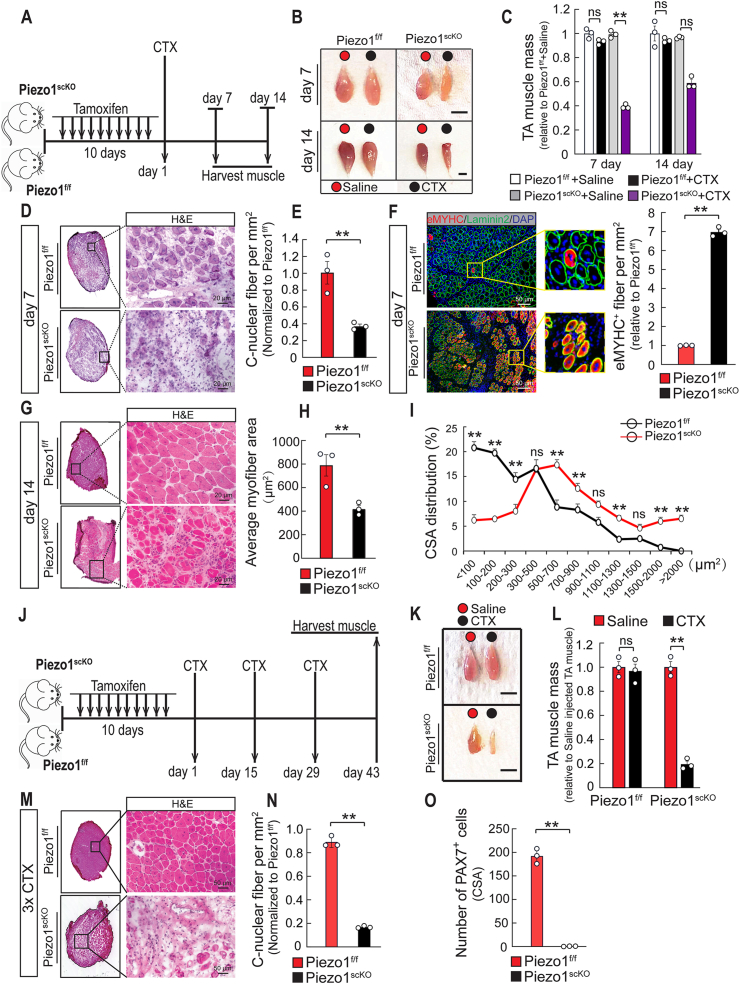
***Piezo1* is required for skeletal muscle regeneration in mice. (A)** Schematic outline of the experimental design for (B–I). **(B)** Macroscopic images of regenerating TA muscles from *Piezo1*^*f/f*^ and *Piezo1*^*scKO*^ mice 7 and 14 days after injury. **(C)** Weights of TA muscles after CTX-induced muscle injury. All the values are relative to *Piezo1*^*f/f*^ + saline. TA muscle weights were normalized to body weights (one way-ANOVA-test: **p < 0.01; ns, *p >* 0.05, n = 3, 2-months-old male mice, 3 sections per sample). **(D)** H&E staining of muscle sections from injured TA muscles of *Piezo1*^*f/f*^ and *Piezo1*^*scKO*^ mice 7 days after CTX injection. **(E)** Numbers of centralized myofibers (C-nuclear fibers) in TA muscles of *Piezo1*^*scKO*^ mice compared to *Piezo1*^*f/f*^ mice (*t*-test: **p < 0.01, n = 3, 2-months-old male mice, 3 sections per sample). **(F)** Immunofluorescence staining for embryonic myosin heavy chain, laminin-2 (green) and DAPI (blue) on TA muscle sections from *Piezo1*^*f/f*^ and *Piezo1*^*scKO*^ mice 7 days after CTX injection. Quantifications are on the right (*t*-test: **p < 0.01, n = 3, 2-months-old male mice, 3 sections per sample). **(G)** H&E staining CTX-injected TA muscles from *Piezo1*^*f/f*^ and *Piezo1*^*scKO*^ (lower) mice 14 days after injury. **(H)** Quantification of average myofiber areas in CTX-injected TA muscles from *Piezo1*^*f/f*^ and *Piezo1*^*scKO*^ mice 14 days after injury (*t*-test: **p < 0.01, n = 3, 2-months-old male mice, 3 sections per sample). **(I)** Distribution of myofibers with different cross-sectional areas (CSA) in TA muscles of *Piezo1*^*f/f*^ and *Piezo1*^*scKO*^ mice 14 days after injury (One-way ANOVA-test: **p < 0.01; ns, *p >* 0.05, n = 3, 2-months-old male mice, 3 sections per sample). **(J)** Schematic outline of the experimental design for (K–O–I). **(K)** Macroscopic images of regenerating TA muscles of *Piezo1*^*f/f*^ and *Piezo1*^*scKO*^ mice after 3 consecutive CTX-induced muscle injuries at day 43. **(L)** Weights of *Piezo1*^*f/f*^ and *Piezo1*^*scKO*^ TA muscles after 3 consecutive CTX-induced muscle injuries at day 43. All values are relative to saline-injected TA muscle of *Piezo1*^*f/f*^ or *Piezo1*^*scKO*^ mice. TA muscle weights were normalized to body weights (One-way ANOVA-test: **p < 0.01; ns, *p >* 0.05, n = 3, 2-months-old male mice, 3 sections per sample). **(M)** H&E staining of non-injured and cardiotoxin-injected TA muscles from *Piezo1*^*f/f*^ and *Piezo1*^*scKO*^ mice after 3 consecutive injections of CTX. **(N)** Quantification of C-nuclear fibers in *Piezo1*^*scKO*^ compared to *Piezo1*^*f/f*^ muscles after 3 consecutive CTX-induced injuries (*t*-test: **p < 0.01; n = 3, 2-months-old male mice, 3 sections per sample). **(O)** Quantification of PAX7^+^ MuSCs on TA muscle sections from *Piezo1*^*f/f*^ and *Piezo1*^*scKO*^ mice after 3 consecutive CTX-induced injuries (*t*-test: **p < 0.01, n = 3, 2-months-old male mice, 3 sections per sample). (For interpretation of the references to color in this figure legend, the reader is referred to the Web version of this article.)

The precocious activation of in *Piezo1*^*scKO*^ MuSC, reduction of MuSC numbers, as well as their reduced proliferation and differentiation strongly suggested that absence of *Piezo1* compromises skeletal muscle regeneration. To address this hypothesis, we inactivated *Piezo1* in adult MuSCs by 10-times injections of TAM and subjected control and *Piezo1*^*scKO*^ TA muscles to cardiotoxin (CTX) injury and subsequent analysis at 7 and 14 days (Fig. 4A). *Piezo1*^*scKO*^ TA muscles showed a strong reduction of TA muscle weight at 7 and 14 days after CTX injury, while CTX-injected *Piezo1*^*f/f*^ control and non-injured *Piezo1*^*scKO*^ TA muscles regained essentially the same mass as non-injured *Piezo1*^*f/f*^ control TA muscles (Fig. 4B and C).

Histological analysis at day 7 after injury revealed reduced formation of regenerated centronuclear fibers in *Piezo1*^*scKO*^ mice compared to *Piezo1*^*f/f*^ control mice (Fig. 4D and E). The relative number of eMYHC^+^ (Myh3) regenerating myofibers was higher in *Piezo1*^*scKO*^ mice than in control mice, indicating delayed formation of myofibers (Fig. 4F). The average fiber cross-sectional area (CSA) area was still reduced by approximately 50% in *Piezo1*^*scKO*^ compared to control mice 14 days after CTX-induced injury (Fig. 4G and H; Fig. S5A). Most regenerated fibers were unusually small 7 and 14 days after injury in *Piezo1*^*scKO*^ mice (<200 μm^2^) and large fibers were missing (Fig. 4I, Fig. S5B).

To characterize the regenerative capacity of *Piezo1*^*scKO*^ mice after repetitive injury, we subjected TA muscles to 3 rounds of CTX-induced muscle regeneration (Fig. 4J). Repetitive muscle injury nearly abolished regeneration in *Piezo1*^*scKO*^ mice with very few newly forming centronuclear myofibers and a severe reduction of muscle mass 14 days after the last injury (Fig. 4K-O; Figs. S5C and D). *Piezo1*^*f/f*^ and *Piezo1*^*scKO*^ TA muscles did not show any significant morphological differences during the course of the experiment without CTX-induced muscle injury (Figs. S5E and F). The very strong muscle regeneration defect was associated with a nearly complete loss of PAX7^+^ MuSCs in *Piezo1*^*scKO*^ mice (Fig. 4O; Fig. S5G), probably caused by compromised self-renewal and the inability of *Piezo1*^*scKO*^ MuSCs to re-acquire quiescence. These data unequivocally establish a major role of *Piezo1* for regeneration of skeletal muscles.

### 2.5 Loss of *Piezo1* enhances ROS and P53 accumulation and leads to increased senescence and cell death of MuSCs

**Fig. 5:**
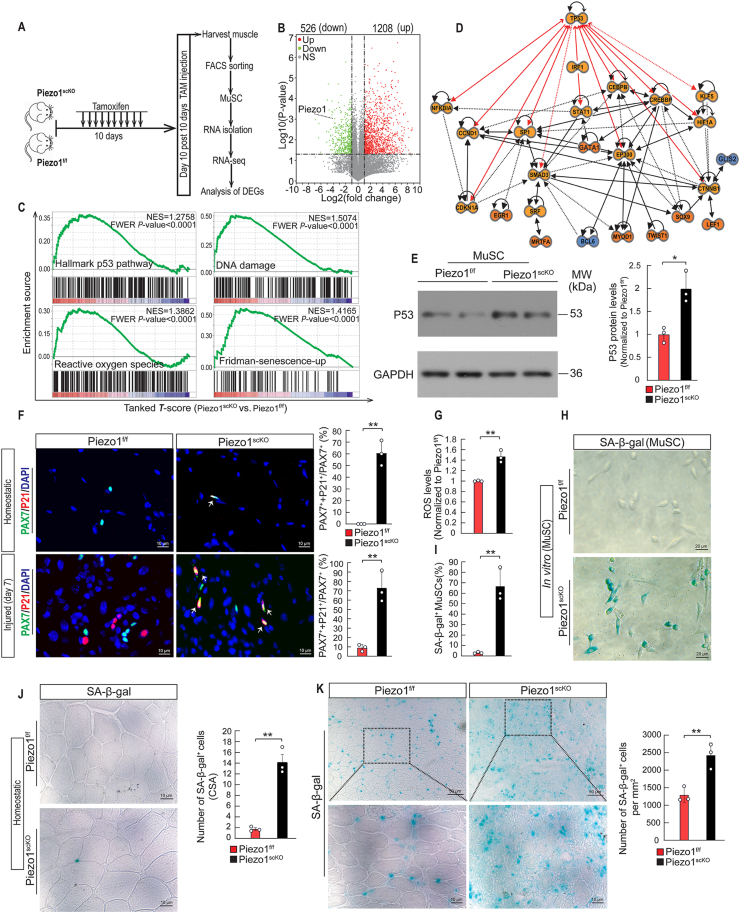
***Piezo1* prevents senescence of MuSCs by reducing ROS levels and P53 accumulation. (A)** Schematic outline of the experimental design. **(B)** Volcano plot depicting DEGs in freshly isolated *Piezo1^f/f^* and *Piezo1*^*scKO*^ MuSC. Red dots represent upregulated and blue dots downregulated genes in *Piezo1*^*scKO*^ MuSCs. Y-axis denotes − log10 *P* values while X-axis shows log2-fold changes. **(C)** Gene set enrichment analysis (GSEA) of 4 different gene sets obtained from MSigDB. **(D)** IPA transcription factors analysis. The network indicates changes of activities of enriched transcription regulators (yellow represents increase and blue color represents decrease of activity) and the known links of transcription factors (red lines indicated direct putative connections of P53). **(E)** Western blots analysis of P53 in freshly isolated *Piezo1*^*f/f*^ and *Piezo1*^*scKO*^ MuSCs. Quantification is shown on the right (*t*-test: *p < 0.05, n = 3, 2-months-old male mice, 3 sections per sample). **(F)** Immunofluorescence staining for P21 (red), PAX7 (green) and DAPI (blue) on TA muscle sections of *Piezo1*^*f/f*^ and *Piezo1*^*scKO*^ mice during homeostasis and 7 days after muscle injury. Quantification is shown on the right (*t*-test: **p < 0.01, n = 3, 2-months-old male mice, 3 sections per sample). **(G)** Measurement of intracellular ROS concentration in isolated *Piezo1*^*f/f*^ and *Piezo1*^*scKO*^ MuSCs (*t*-test: **p < 0.01, n = 3, 2-months-old male mice). **(H)** SA-β-gal staining of *Piezo1*^*f/f*^ and *Piezo1*^*scKO*^ MuSCs to detect senescent cells. **(I)** Quantification of SA-β-gal signals from (H). At least 500 cells per individual sample were counted (*t*-test: **p < 0.01; n = 3, 2-months-old male mice). **(J)** SA-β-gal staining of TA muscle sections from *Piezo1*^*f/f*^ and *Piezo1*^*scKO*^ during homeostasis and **(K)** 7 days after muscle injury. Quantifications are on the right (*t*-test: **p < 0.01, n = 3, 2-months-old male mice, 3 sections per sample). (For interpretation of the references to color in this figure legend, the reader is referred to the Web version of this article.)

To study the molecular mechanism enabling Piezo1-dependent regulation of MuSC quiescence, skeletal muscle regeneration, and MuSC maintenance, we subjected FACS-isolated MuSCs from *Piezo1*^*f/f*^ and *Piezo1*^*scKO*^ mice to RNAseq analysis (Fig. 5A). We identified 1734 differently expressed genes (DEGs) between *Piezo1*^*f/f*^ and *Piezo1*^*scKO*^ MuSC. 1208 genes were upregulated in *Piezo1*^*scKO*^ MuSC, while 526 were downregulated based on absolute fold-changes ≥2 (Benjamini-Hochberg-corrected *p*-value ≤ 0.05) (Fig. 5B). Gene set enrichment analysis (GSEA) revealed differential expression of genes related to cell senescence in *Piezo1*^*scKO*^ MuSC, including DNA damage response, increase of ROS production, lysosome activity, and telomerase dysfunction (Fig. 5C; Fig. S6). Furthermore, Metacore and Ingenuity Pathway Analysis (IPA) indicated that changes in the activity of enriched transcriptional regulators can be traced back to P53 (Fig. 5D). These data suggested that P53 may play a key role in mediating effects in the regulatory network downstream of Piezo1. To validate this assumption, we performed western blot analysis, which revealed a strong accumulation of P53 in *Piezo1*^*scKO*^ MuSCs (Fig. 5E). Furthermore, we detected increased expression of P21, a canonical target of P53, in undamaged and regenerating *Piezo1*^*scKO*^ muscles (Fig. 5F). Increased production of ROS promotes accumulation of P53 in numerous cell types [37,38], which sparked the idea that the enhanced presence of P53 in *Piezo1*^*scKO*^ MuSCs is due to increased ROS production. Measurement of ROS confirmed increased ROS production in *Piezo1*^*scKO*^ MuSCs (Fig. 5 G), while treatment of MuSCs with the Piezo1-activator Yoda1 had no effects on ROS-levels in WT MuSCs (Fig. S7A). Furthermore, we detected a strong increase of oxidative DNA damage as indicated by accumulation of 8-oxoG (Fig. S7B) and increased numbers of MuSCs with the DNA-damage marker γH2AX in *Piezo1*^*scKO*^ muscles, both in undamaged and in regenerating conditions (Fig. S7C). To analyze whether increased ROS production due to loss of *Piezo1* is indeed responsible for accumulation of P53, we treated *Piezo1*^*scKO*^ MuSCs with the ROS scavenger NAC. We observed that scavenging of ROS normalized P53 to control levels (Figs. S7D and E), indicating that enhanced ROS production is responsible for P53 accumulation in *Piezo1*^*scKO*^ MuSC.

DNA damage and accumulation of P53 often results in apoptosis and cellular senescence [39]. To explore whether loss of *Piezo1* in MuSCs and the resulting increase in ROS and P53 levels increase the number of apoptotic and senescent MuSCs, we stained for senescence-associated β-galactosidase (SA-β-Gal), a commonly accepted marker for senescent cells [40]. The number of SA-β-Gal^+^ MuSCs was dramatical higher in cultured *Piezo1*^*scKO*^ MuSCs compared to controls (Fig. 5H and I). Treatment of cultured WT MuSCs with the Piezo1 channel activator Yoda1 did not reduce the number of senescent cells, probably because senescence is very low at baseline conditions (Fig. S8). We also found a strong increase of senescent MuSCs in muscle tissue sections from *Piezo1*^*scKO*^ compared to *Piezo1*^*f/f*^ control mice (Fig. 5J; Fig. S9A), which was even more evident in regenerating TA muscles 7 days after injury (Fig. 5K; Fig. S9B), suggesting that mechanical cues sensed by Piezo1 suppress cellular senescence of MuSC. Moreover, we observed a strong increase of apoptosis in cultured MuSC, in which the floxed Piezo1 alleles were deleted by Ad-Cre infection, based on TUNEL- and cleaved Casp3-staining (Fig. S10).

### 2.6 Inactivation of *Piezo1* increases expression of T-Type Ca^2+^ channels, elevates Ca^2+^ influx, and stimulates ROS production in MuSCs

**Fig. 6:**
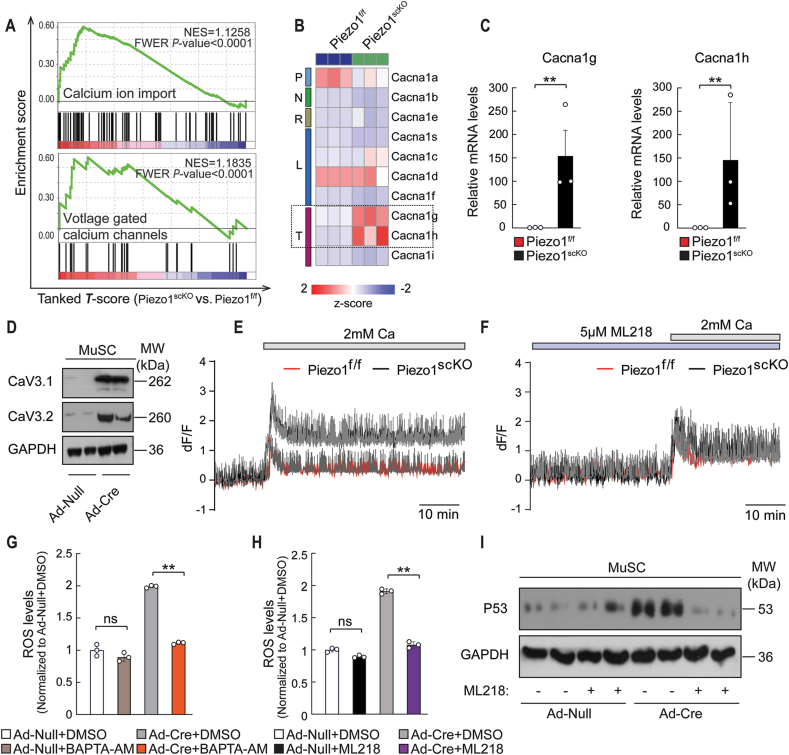
**Inactivation of Piezo1 increases expression of T-type Ca**^**2+**^**channels and Ca**^**2+**^**influx in MuSC, elevating ROS production. (A)** GSEA for “calcium ion import” and “voltage gated calcium channels” based on RNAseq of *Piezo1*^*f/f*^ and *Piezo1*^*scKO*^ MuSCs. **(B)** Gene expression heatmap based on RNAseq of *Piezo1*^*f/f*^ and *Piezo1*^*scKO*^ MuSC. **(C)** RT-qPCR analysis of *Cacna1g and Cacna1h* expression in *Piezo1*^*f/f*^ and *Piezo1*^*scKO*^ MuSCs. mRNA levels were normalized to *Gapdh* (*t*-test: **p < 0.01, n = 3, 2-months-old male mice). **(D)** Western blot analysis of CaV3.1 and CaV3.2 in *Piezo1*^*f/f*^ and *Piezo1*^*scKO*^ MuSCs. **(E)** Fura-4-AM Ca^2+^influx measurements in cultured *Piezo1*^*f/f*^ and *Piezo1*^*scKO*^ MuSCs in presence (2 mM Ca^2+^) and absence (0 mM Ca^2+^) of extracellular Ca^2+^. **(F)** Ca^2+^ influx measurements of ML218 treated *Piezo1*^*f/f*^ and *Piezo1*^*scKO*^ MuSCs. **(G)** Intracellular ROS concentration of Ad-Cre or Ad-null virus infected *Piezo1*^*ff*^ MuSCs after treatment with 15 μM BAPTA-AM for 24h (One-way ANOVA-test: **p < 0.01; ns, *p >* 0.05, n = 3 independent experiments, 6 replicates per experiment. **(H)** Intracellular ROS concentration in Ad-Cre or Ad-null virus infected *Piezo1*^*f/*f^ MuSCs after treatment with 5 μM ML218 for 48h (One-way ANOVA-test: **p < 0.01; ns, *p >* 0.05, n = 3 independent experiments, 6 replicates per experiment). **(I)** Western blot analysis of P53 in Ad-Cre or Ad-null virus infected *Piezo1*^*f/*f^ MuSCs after treatment with 5 μM ML218 for 48h.

The increase of ROS levels in *Piezo1*^*scKO*^ MuSCs was surprising, since formation of ROS is usually enhanced by increased but not by decreased Ca^2+^ levels [41]. In *Piezo1*^*scKO*^ MuSCs we expected to the see reduced Ca^2+^ levels and thus reduced ROS production, because Piezo1 allows Ca^2+^ influx in response to different types of external forces [42]. Therefore, we decided to have a closer look at Ca^2+^ influx and molecules involved in this process. GSEA of *Piezo1*^*scKO*^ MuSCs revealed a strong enrichment of genes responsible for Ca^2+^ import, including voltage-gated Ca^2+^ channels (Fig. 6A). Further analysis of RNAseq data uncovered increased expression of the voltage-dependent T-Type Ca^2+^ channel genes *Cacna1g* and *Cacna1h* in freshly isolated *Piezo1*^*scKO*^ compared to *Piezo1*^*f/f*^ MuSCs (Fig. 6B), which was validated by RT-PCR experiments (Fig. 6C). In addition, we observed a strong elevation of the corresponding CaV3.1 (*Cacna1g*) and CaV3.2 (*Cacna1h*) channels in MuSCs, in which the floxed Piezo1 alleles were deleted by Ad-Cre infection (Fig. 6D), strongly suggesting that loss of the Piezo1 Ca^2+^ channel leads to a compensatory increase of T-Type Ca^2+^ channels. To investigate whether the strong increase of CaV3.1 and CaV3.2 also increases intracellular Ca^2+^ levels, we performed Ca^2+^ influx measurements in cultured *Piezo1*^*scKO*^ and *Piezo1*^*f/f*^ MuSCs using the Ca^2+^ -sensitive Fura-4-AM dye [29]. We detected a clear increase of Ca^2+^ influx in *Piezo1*^*scKO*^ compared to *Piezo1*^*f/f*^ MuSCs (Fig. 6E), which was normalized by addition of ML218, a specific inhibitor of T-Type Ca^2+^ channels [43] (Fig. 6F).

Next, we investigated whether the increased Ca^2+^ influx is indeed responsible for enhanced ROS production in *Piezo1*^*scKO*^ MuSCs. To this end, we measured ROS levels in MuSCs, in which the floxed Piezo1 alleles were deleted by Ad-Cre infection, either in the absence or presence of the Ca^2+^ chelator BAPTA-AM. Administration of BAPTA-AM reduced ROS in *Piezo1*^*scKO*^ MuSCs back to control levels while no decrease was observed in *Piezo1*^*f/f*^ MuSCs (Fig. 6G). Strikingly, we found the same normalization of ROS levels, when *Piezo1*^*scKO*^ MuSCs were treated with ML218 (Fig. 6H). To further prove the critical role of T-Type Ca^2+^ channel-mediated Ca^2+^ influx, we measured P53 levels, which are increased in *Piezo1*^*scKO*^ MuSCs due to raised ROS concentrations. We found that administration of ML218, lowered P53 in Piezo1-mutant MuSCs to control levels (Fig. 6I), confirming that the compensatory increase of Ca^2+^ influx via T-Type Ca^2+^ channels raises ROS levels and P53 expression.

### 2.7 Increased Ca^2+^ influx via T-Type Ca^2+^ channels after inactivation of *Piezo1* activates cPKC, thereby stimulating NOX4 expression and ROS generation

**Fig. 7:**
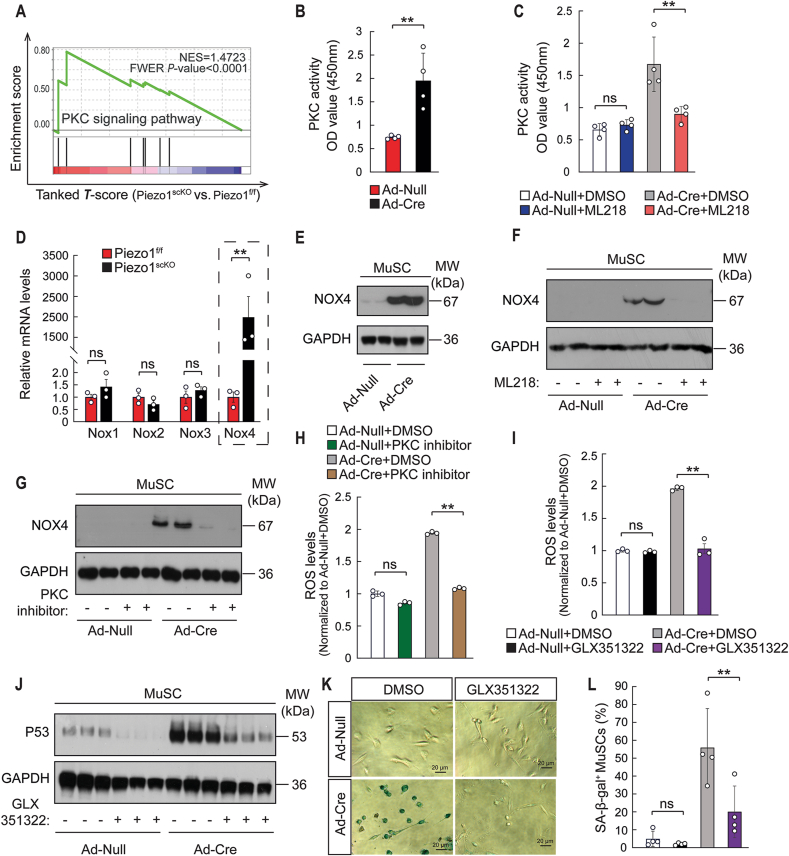
**Inactivation of Piezo1 stimulates expression of Nox4 via the Ca**^**2+**^**-PKC signaling axis. (A)** GSEA for “PKC signaling pathway” based on RNAseq of *Piezo1*^*f/f*^ and *Piezo1*^*scKO*^ MuSC. **(B)** PKC activity in Ad-Cre or Ad-null virus infected *Piezo1*^*f/*f^ MuSCs (*t*-test: **p < 0.01, n = 4 independent experiments, 8 replicates for each experiment). **(C)** PKC activity in Ad-Cre or Ad-null virus infected *Piezo1*^*f/*f^ MuSCs after treatment with 5 μM ML218 for 48h (One-way ANOVA-test: **p < 0.01; ns, *p >* 0.05, n = 4 independent experiments, 8 replicates per experiment). **(D)** RT-qPCR analysis of *Nox1, Nox2, Nox3, and Nox4* expression in *Piezo1*^*f/f*^ and *Piezo1*^*scKO*^ MuSCs. mRNA levels were normalized to *Gapdh* SEM (*t*-test: **p < 0.01; ns, *p >* 0.05, n = 3, 2-months-old male mice). **(E)** Western blot analysis of NOX4 in Ad-Cre or Ad-null virus infected *Piezo1*^*f/*f^ MuSCs. **(F)** Western blot analysis of NOX4 in Ad-Cre or Ad-null virus infected *Piezo1*^*f/*f^ MuSCs after treatment with 5 μM ML218 for 48h. **(G)** Western blot analysis of NOX4 in Ad-Cre or Ad-null virus infected *Piezo1*^*f/*f^ MuSCs after treatment with 10 nM pan-PKC inhibitor (Go 6983) for 48h. **(H)** Intracellular ROS concentration in Ad-Cre or Ad-null virus infected *Piezo1*^*f/*f^ MuSCs after treatment with 10 nM pan-PKC inhibitor (Go 6983) for 48h (One-way ANOVA-test: **p < 0.01; ns, *p >* 0.05, n = 3 independent experiments, 6 replicates for each experiment). **(I)** Intracellular ROS concentration in Ad-Cre or Ad-null virus infected *Piezo1*^*f/*f^ MuSCs after treatment with 5 μM GLX351322 for 48h (One-way ANOVA-test: **p < 0.01; ns, *p >* 0.05, n = 3 independent experiments, 6 replicates for each experiment). **(J)** Western blot analysis of P53 in Ad-Cre or Ad-null virus infected *Piezo1*^*f/*f^ MuSCs after treatment with 5 μM GLX351322 for 48h. **(K)** SA-β-gal staining of Ad-Cre or Ad-null virus infected *Piezo1*^*f/*f^ MuSCs after 48h treatment with GLX351322 or DMSO. **(L)** Quantifications of SA-β-gal^+^ MuSCs of Ad-Cre or Ad-null virus infected *Piezo1*^*f/*f^ MuSCs after treatment with 5 μM GLX351322 for 48h. (One-way ANOVA-test: **p < 0.01; ns, *p >* 0.05, n = 4 independent experiments, 10 repeats for each experiment, 30 fields chosen randomly were quantified for each repeat).

Although increased Ca^2+^ concentrations are known to enhance the activity of ROS-generating enzymes, the strong increase in ROS levels in in *Piezo1*^*scKO*^ MuSCs suggested an additional mechanism. Ca^2+^ binds to the C2-domain of conventional PKCs (cPKCs) to induce translocation of cPKCs to the plasma membrane, which enables binding of DAG and phosphatidylserines, classical activators of cPKCs [44]. GSEA of *Piezo1*^*scKO*^ MuSCs disclosed enhanced activity of the PKC signaling pathway (Fig. 7A), confirmed by higher enzyme activity of PKC in cultured MuSC, in which the floxed Piezo1 alleles were deleted by Ad-Cre infection (Fig. 7B). Normalization of PKC enzyme activity in *Piezo1*-deficient MuSCs by the T-Type Ca^2+^ channel blocker ML218 confirmed that the increased PKC activity was induced by elevated Ca^2+^ influx mediated via CaV3.1 and CaV3.2 (Fig. 7C). Since PKC was recently reported to activate expression of the ROS-generating enzyme NOX4 in kidney cells [45], we investigated the expression of different NOX enzymes in MuSC. We found that increased PKC activity in *Piezo1*-deficient MuSCs correlated with increased gene expression of *NOX4* but not of *NOX1*, *NOX2* and *NOX3* (Fig. 7D). Furthermore, western blot analysis revealed strongly elevated levels of NOX4 in MuSCs after inactivation of *Piezo1* (Fig. 7E), which was completely normalized by the T-Type Ca^2+^ channel blocker ML218 (Fig. 7F). To learn whether activation of cPKC is indeed instrumental for elevated ROS levels in *Piezo1*^*scKO*^ MuSCs, we inhibited cPKC activity pharmacologically. Addition of Go 6983, a pan-PKC inhibitor, strongly reduced NOX4 expression (Fig. 7G) and also normalized ROS in cultured Piezo1-deficient MuSCs to control levels (Fig. 7H) [46]. We also found that inhibition of NOX4 with GLX351322 reduced ROS in cultured Piezo1-deficient MuSCs to control levels, abolished increased P53 expression in Piezo1-deficient MuSCs (Fig. 7I and J) and prevented cellular senescence (Fig. 7K and L). Taken together, the data indicate that increased Ca^2+^ influx via T-Type Ca^2+^ channels in *Piezo1*-deficient MuSCs activates cPKC, which stimulates NOX expression, raises ROS and P53 levels, and induces cellular senescence.

### 2.8 Pharmacological inhibition of P53 reduces formation of senescent MuSCs and improves muscle regeneration in *Piezo1*^*scKO*^ mice

**Fig. 8:**
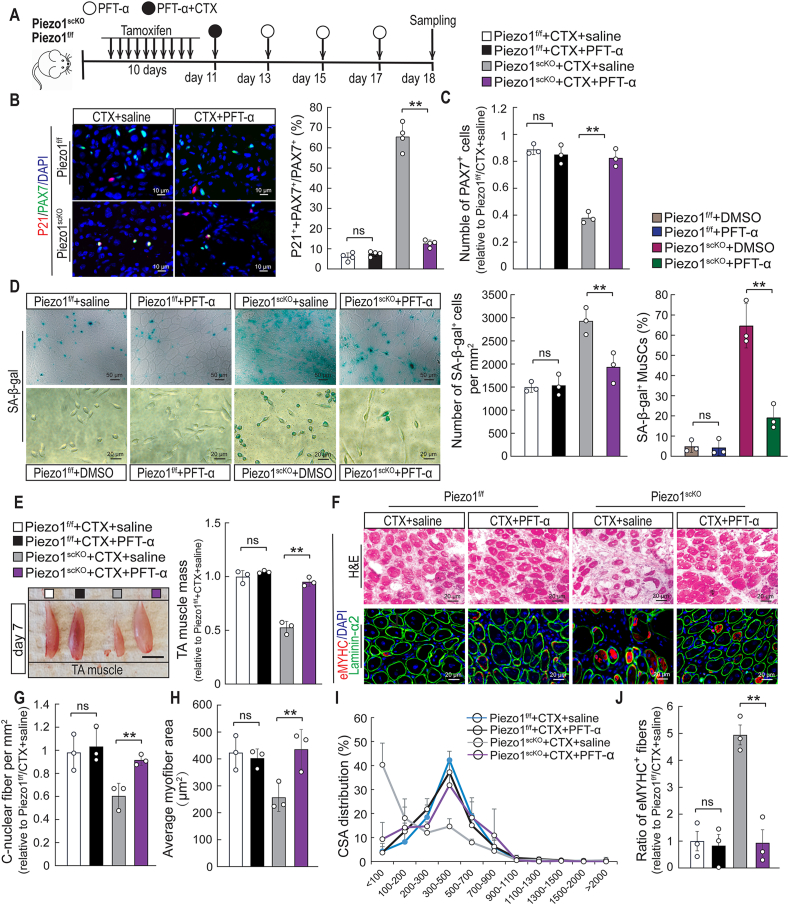
**Inhibition of P53 prevents accumulation of senescent MuSCs in *Piezo1***^***scKO***^**mice and partially rescues skeletal muscle regeneration. (A)** Schematic outline of the experimental design. **(B)** Immunofluorescence staining for P21(red) and PAX7 (green) and DAPI (blue) on TA muscle sections from *Piezo1^f/f^* and *Piezo1*^*scKO*^ mice after PFT-α and saline treatment, 7 days after CTX injections. Quantification is shown on the right (One-Way ANOVA-test: **p < 0.01; ns, *p >* 0.05, n = 4, 2-months-old male mice, 3 sections per sample). **(C)** Number of PAX7^+^ MuSCs on TA muscle sections from *Piezo1*^*f/f*^ and *Piezo1*^*scKO*^ mice after PFT-α and saline treatment, 7 days after CTX injections (One-Way ANOVA-test: **p < 0.01; ns, *p >* 0.05, n = 3, 2-months-old male mice, 10 sections per sample). **(D)** SA-β-gal staining of TA muscle sections from *Piezo1*^*f/f*^ and *Piezo1*^*scKO*^ mice after PFT-α and saline treatment, 7 days after CTX injections. Lower panel: SA-β-gal staining of MuSCs from *Piezo1*^*f/f*^ and *Piezo1*^*scKO*^ mice after 5 days *in vitro* treatment with PFT-α or DMSO. Quantification of SA-β-gal^+^ cells on sections (One-Way ANOVA-test: **p < 0.01; ns, *p >* 0.05, n = 3, 2-months-old male mice, 10 sections per sample) and of MuSCs in culture (*t*-test: **p < 0.01; n = 3 independent experiments, 10 replicates per experiment. ≥500 cells per replicate) are shown on the right. **(E)** Macroscopic images of *Piezo1*^*f/f*^ and *Piezo1*^*scKO*^ TA muscles after treatment with PFT-α and saline, 7 days after injury. TA muscle weights are shown on the right. All values are relative to saline-injected *Piezo1*^*f/f*^ mice. TA muscle weights were normalized to body weights (One-way ANOVA-test: **p < 0.01; ns, *p >* 0.05, n = 3, 2-months-old male mice, 3 sections per sample). **(F)** H&E staining of *Piezo1*^*f/f*^ and *Piezo1*^*scKO*^ TA muscles after mice after PFT-α and saline treatment, 7 days after injury. Lower panel: immunofluorescence staining for eMYHC (*Myh3*, red), laminin-⍺2 (green) and DAPI (blue). **(G, H)** Number of C-nuclear fibers (I) and average myofiber area (J) of *Piezo1*^*f/f*^ and *Piezo1*^*scKO*^ TA muscles after treatment with PFT-α and saline, 7 days after injury (One-Way ANOVA-test: **p < 0.01; ns, *p >* 0.05, n = 3, 2-months-old male mice, 3 sections per sample). **(I)** Distribution of myofiber cross-sectional areas (CSA) in TA muscles of *Piezo1*^*f/f*^ and *Piezo1*^*scKO*^ mice after PFT-⍺ and saline treatment, 7 days after muscle injury. **(J)** Quantification of eMYHC (*Myh3*) staining shown in (F, lower panel) (One-Way ANOVA-test: **p < 0.01; ns, *p >* 0.05, n = 3, 2-months-old male mice, 10 sections per sample). (For interpretation of the references to color in this figure legend, the reader is referred to the Web version of this article.)

The strong accumulation of P53 and P21 in *Piezo1*^*scKO*^ MuSCs suggested a causal role in the formation of senescent MuSCs, which might be the underlying reason for defective skeletal muscle regeneration. To explore this hypothesis, we treated *Piezo1*^*scKO*^ and *Piezo1*^*f/f*^ control mice during muscle regeneration with the P53 inhibitor Pifithrin-α (PFT-α) (Fig. 8A). Immunofluorescence staining 7 days after injection of CTX showed a strong reduction of the numbers of P21^+^ MuSCs in *Piezo1*^*scKO*^ mice treated with PFT-α, indicating successful inhibition of P53 activity (Fig. 8B). Likewise, we observed a strong decline of γH2AX^+^ MuSCs in *Piezo1*^*scKO*^ mice after PFT-α treatment, while no significant changes were observed in *Piezo1*^*f/f*^ control mice with or without PFT-α treatment (Fig. S11). Furthermore, we observed that the PFT-α treatment not only reduced the number of P21^+^ and γH2AX^+^ MuSCs but also prevented loss of PAX7^+^ MuSCs in Piezo1^scKO^ muscles (Fig. 8C, Fig. S11), indicating that loss of MuSCs in *Piezo1*^*scKO*^ muscles is initiated by aberrant activation of P53.

Next, we investigated whether inhibition of increased P53 activity in *Piezo1*^*scKO*^ MuSCs prevents accumulation of senescent MuSCs and restores muscle regeneration. Strikingly, PFT-α treatment strongly reduced the number of SA-β-Gal^+^ cells in *Piezo1*^*scKO*^ muscles, essentially matching the numbers found in *Piezo1*^*f/f*^ control muscles (Fig. 8D). Similar results were obtained when isolated MuSCs from *Piezo1*^*f/f*^ and *Piezo1*^*scKO*^ mice were treated for 5 days with PFT-α (Fig. 8D). Furthermore, treatment with PFT-α also reestablished muscle regeneration in *Piezo1*^*scKO*^ mice. We detected a major increase of TA muscle mass in PFT-α-treated versus untreated *Piezo1*^*scKO*^ mice 7 days after injury (Fig. 8E). The number of newly formed centronuclear myofibers increased dramatically as well, essentially eradicating the presence of unrepaired areas in damaged muscles of *Piezo1*^*scKO*^ mice (Fig. 8F). The increase of newly formed centronuclear myofibers went along with a decrease of smaller fibers and an increase of larger fibers as well as disappearance of eMYHC-positive myofibers, all indicative of improved skeletal muscle regeneration (Fig. 8G–J). Taken together, we concluded that Piezo1-mediated suppression of ROS production and P53 accumulation is critical to prevent formation of senescent MuSC, thus maintaining a healthy MuSC pool allowing efficient skeletal muscle regeneration.

## 3 Discussion

MuSCs are situated on contractile myofibers and therefore directly exposed to mechanical signals, resulting from muscle tonus and muscle contractions. Several studies have investigated the impact of mechanical signaling on MuSCs [47,48], but knowledge about the mechanisms by which MuSCs sense mechanical cues is very limited. In this study, we uncovered that the mechanosensitive ion channel Piezo1 is highly expressed in MuSCs, prevents precocious activation of MuSC, and is required for regular MuSC proliferation and differentiation. Furthermore, we demonstrate that loss of *Piezo1* increases ROS formation and promotes accumulation of P53, resulting in massive induction of senescence, depletion of the MuSC pool, and eventually in severe defects of skeletal muscle regeneration. Since MuSCs undergo enhanced senescence during aging when physical activity and muscle tonus decline, we speculate that mechanosensing by Piezo1 plays an important role to maintain skeletal muscle homeostasis and to prevent senescence of MuSCs in geriatric muscles.

Quiescence of MuSCs is regulated by numerous signaling pathways, which exert their functions within the MuSC niche, in which the myofiber acts as the primary signal-providing hub. Ligands provided by muscle fibers include delta-like 1 (DLL1), WNT4, Oncostatin M (OSM), and many more (see Ref. [49] for a recent review). In addition, other cells within in the MuSC niche, such as endothelial cells and fibroadipogenic precursor cells (FAPs), support MuSCs to maintain quiescence. Quiescence was initially assumed to reflect a default state of cellular inactivity, but mounting evidence indicates that MuSCs require active input to regulate quiescence, which is pivotal to guarantee stemness of MuSCs and to maintain regenerative functions. It is surprising that mechanosensing via the Piezo1 channel upholds quiescence instead of activating MuSC, since accretion of MuSCs by myofibers is required for sustained hypertrophic growth of myofibers following enhanced mechanical load, despite some controversy about the role of MuSCs for skeletal muscle hypertrophy [50,51]. Apparently, muscles have developed mechanisms to distinguish between strong mechanical load, resulting in activation of MuSCs and hypertrophic growth, and low-level tonic signals, preventing activation of MuSCs. Our study uncovers that active Piezo1 is crucial for arresting MuSCs in quiescence. The biological meaning of mechanosensing for keeping MuSCs in quiescence is currently unclear but it is tempting to speculate that a decline of low-level tonic signaling due to diminished force generation by myofibers awakens MuSCs, allowing renewal of myofibers to regain strength. Similarly, continuously reduced mechanosensing in geriatric muscles with lowered muscle tonus may result in permanent activation of muscle stem cells in an attempt to restore muscle fiber strength but eventually causing depletion of the MuSC pool. The increased propensity of MuSCs in geriatric muscle for activation and differentiation as well as the reduction of MuSC numbers during aging supports such a hypothesis [22].

MuSCs undergo enhanced senescence in muscles of geriatric mice, which is associated with impaired muscle regeneration after injury [23]. Senescence-induced impairment of muscle regeneration during aging or in progeria models has been explained by persistent P38 MAPK activity, downregulation of Slug, a member of the Slug/Snail superfamily of zinc-finger transcriptional factor, downregulation of the cell surface protein CDON [52], reduced mitophagy, TGF-β–induced SMAD3 activation, and over-activation of Notch [53,54], all promoting stem cell exhaustion by increased senescence [22,23,55]. The current study extends this list and establishes Piezo1-mediated mechanosensing as an important suppressor of MuSC senescence in skeletal muscles. However, we currently do not know whether expression of *Piezo1* or Piezo1-mediated mechanosensing declines during aging, although the lowered muscle tonus in aged muscles makes such a scenario likely. Loss of *Piezo1* in our experimental model has some similarities to P53-dependent senescence in activated Numb-deficient MuSCs during muscle regeneration, resulting in regeneration defects of skeletal muscles [56]. In both cases, MuSC senescence relies on P53 activation but in contrast to Numb-deficient MuSC, the absence of *Piezo1* instigates MuSC senescence under homeostatic conditions without prior injury. Increased senescence of MuSCs alone may be sufficient to explain depletion of the MuSC pool and regeneration defects but the loss of quiescence will certainly contribute. Two different scenarios seem possible: (i) Loss of *Piezo1* directly initiates senescence of MuSCs via increased ROS-production and P53 activation, which leads to loss of MuSC. (ii) Loss of *Piezo1* causes aberrant activation of MuSC, which subsequently increases ROS-production and P53 activation. Single cell RNA-seq of MuSCs after *in situ* fixation to avoid artifacts resulting from activation of MuSCs during the isolation procedure may help to answer the question whether senescence-specific gene are already expressed in MuSCs of *Piezo1*^*scKO*^ mice prior to isolation [57].

Our study uncovered the basic mechanisms by which inactivation of *Piezo1* increases ROS formation and activates P53, although some questions still remain open. We found that inactivation of the Piezo1 Ca^2+^ channel caused up-regulation of the T-Type Ca^2+^ channel genes *Cacna1g* and *Cacna1h*, most likely by a compensatory mechanism. As net result, Ca^2+^ influx into *Piezo1*-deficient MuSCs was higher, which may activate ROS-generating enzymes and formation of free radicals [41]. More importantly, we also detected a Ca^2+^-dependent increase of cPKC activity, which strongly induced expression of the ROS-generating enzyme NOX4, confirming previous results in kidney cells [45]. Since inhibition of NOX4 essentially normalized ROS levels, we conclude that induction of NOX4 is the main reason for elevated ROS production in *Piezo1*-deficient MuSC. Changes in Ca^2+^ concentrations are associated with senescence of skeletal muscle fibers, further supporting a link between aberrant Ca^2+^ handling and senescence but more detailed studies are necessary to understand the precise mechanisms [58].

We found that inhibition of ROS prevents increase of P53 and that inhibition of p53 attenuates senescence of MuSC, partially rescuing the skeletal muscle regeneration defect in *Piezo1*^*scKO*^ mice. These observations clearly suggest that ROS-mediated activation of P53 plays a pivotal role for triggering senescence in *Piezo1*^*scKO*^ MuSC. ROS is well known to activate P53 in different conditions, although it has been debated whether activation of P53 by ROS is solely facilitated through DNA damage response, redox signaling, or both [59]. We observed enhanced levels of the DNA damaged markers 8-oxoG and γH2AX in *Piezo1*^*scKO*^ MuSC, which argues for ROS-induced DNA damage as a critical trigger of P53 activation but we do not want to exclude other ROS-dependent pathways. Moreover, it was reported recently that Piezo1 directly binds to P53, and that knockdown of *Piezo1* upregulates expression of P53 in human esophageal carcinoma cell lines, suggesting mutual interaction based on protein-protein interactions [60]. Since we observed that scavenging of ROS prevents increase of P53, we do not believe that such a mechanism is relevant for increased P53 activity and increased senescence in *Piezo1*^*scKO*^ MuSC.

In conclusion, we demonstrate that Piezo1-mediated mechanosensing is essential for maintaining quiescence of MuSCs and for preventing P53-dependent senescence. Our findings underscore the importance of mechanical signal transduction in skeletal muscle homeostasis and suggest tight interactions between mechanical activities, senescence, and age-related decline of skeletal muscle functions. We assume therapeutic targeting of Piezo1 is an interesting option to prevent age-related loss of MuSCs and improve muscle functions at advanced age.

## 4 Materials and methods

### 4.1 Animal models

The *Piezo1*^*f/f*^ *(Piezo1*^*loxp/loxp*^*)* and *Pax7*^*ICN*^ mouse strains have been described previously [29,61]. The *Pax7*^*CreERT2*^ mouse strain was obtained from The Jackson Laboratory [62]. Primers used for genotyping are shown in Supplementary Table 1. Tamoxifen (Sigma, dissolved in corn oil) was administered intraperitoneally at 75 mg per kg body weight. Muscle injury was induced by injection of 50 μl Cardiotoxin (CTX) (0.06 mg/mL, Sigma) into the TA muscle. All animal experiments were done in accordance with the Guide for the Care and Use of Laboratory Animals published by the US National Institutes of Health (NIH Publication No. 85-23, revised 1996) and according to the regulations issued by the Institutional Animal Care and Use Committee of Sichuan Agricultural University (Chengdu, China).

### 4.2 Pifithrin-α treatment of mice

Pifithrin-α (Pifithrin-α hydrobromide) (1267, Tocris), a pan-P53 inhibitor was used to inhibit P53 activities *in vivo* [63]. Intraperitoneal injections of Pifithrin-α were performed at a concentration of 2 mg/kg of mice body weight together with tamoxifen treatment for 10 consecutive days, prior to CTX injection. Intraperitoneal administration of Pifithrin-α (2.2 mg per 1 kg body weight) was continued every second day after induction of muscle injury until isolation of the samples as indicated.

### 4.3 MuSCs and myofiber cultures

MuSCs were purified via FACS and cultured in growth medium 1 (DMEM GlutaMAX containing 20% FCS 1%, Penicillin/Streptomycin, and 5 ng/mL basic fibroblast growth factor (bFGF)) [6]. The medium was changed at every second day. MuSC differentiation was induced by changing growth to differentiation medium (DMEM GlutaMAX containing 2% horse serum and 1% Penicillin/Streptomycin), after 6 days culturing in growth medium. Myofibers were isolated by enzymatic digestion of isolated FBD muscle with collagenase P (0.02%, Roche), and cultured in growth medium 2 (DMEM medium with 20% fetal calf serum (FCS) and bFGF (5 ng/mL)) [64]. MuSCs or myofibers isolated from WT mice were cultured in the absence or presence of 1.5 μM, 3 μM or 10 μM Yoda1 (SML1558, Sigma) for 8h (Myofibers) or 48h (MuSCs) and then fixed with 4% paraformaldehyde (PFA) for further analysis. FACS purified MuSCs from *Piez1*^*fl/fl*^ mice were cultured in growth medium and infected with Cre recombinase containing Ad-Cre virus (Vector biolabs) for at least 48h to induce Cre-mediated deletion of Piezo1 in MuSCs *in vitro*. MuSCs treated with adenoviruses that do not express Cre (Ad-Null) served as controls.

### 4.4 ROS measurements

To detect total cellular ROS levels, MuSCs isolated from *Piezo1*^*scKO*^ and *Piezo1*^*f/f*^ mice were cultured in growth medium. After five days of culture, cellular ROS levels were measured using a Total ROS detection kit (51011, ENZO) following the manufacturer's instructions. Signals were recorded using a fluorescence plate reader (LB 940, Mithras).

### 4.5 *In vitro* EdU incorporation assay

Growth medium was supplemented by addition of EdU to a final concentration of 10 μM, 3 h before fixation of cultured MuSCs and then analyzed using the Click-iT EdU kit (Invitrogen) according to the manufacturer's protocol.

### 4.6 TUNEL assay

Cells were fixed in 4% PFA for 10 min and then subjected to the TUNEL reaction using the TUNEL Assay Apoptosis Detection Kit (C10246, Invitrogen), following the manufacturer's instructions.

### 4.7 Protein kinase C activity assay

PKC activity in MuSCs was measured with a PKC Kinase Activity Assay Kit (ab139437, Abcam). Cells were washed once with PBS and lysed with lysis buffer containing 50 mM KH2PO4, 1.5 mM MgCl_2_, 10 mM NaCl, and 1 mM EDTA (pH 7.4) on ice. Cells were collected into Eppendorf tubes and centrifuged (18,000×*g*, 15 min, 4 °C). Supernatants were collected and diluted 1:20 using kinase assay dilution buffer provided in the kit. Diluted samples were pipetted to the 96-well plate included in the kit, and processed according to the manufacturer's instructions. Protein concentrations of samples were determined with BCA to normalize PKC activities to the protein concentrations [65].

### 4.8 Immunofluorescent analysis

For immunofluorescence stainging, cells were placed onto Matrigel-coated dishes and fixed with 4% PFA for 10 min. Myofibers were fixed with 2% PFA for 10 min. After permeabilization in 0.1% Triton X-100 for 15 min, samples were blocked in 3% BSA for 30 min and incubated with the following antibodies at 4 °C overnight: anti-PAX7 antibody(Developmental Studies Hybridoma Bank, mouse; 1:500), anti-MF20 antibody (Developmental Studies Hybridoma Bank, mouse; 1:200), anti-Piezo1 antibody (15939-1-AP, Proteintech, rabbit; 1:200), anti-MYOD antibody (ab-64159, Abcam, rabbit; 1:500), anti-laminin-2 antibody (sc-59854, Santa Cruz, rat; 1:500) and anti-CALCR antibody (ab11042, Abcam, rabbit; 1:1000), Anti-Cleaved caspase-3 antibody (9611, Cell signaling, rabbit: 1:400), Anti-Oxoguanine 8 antibody (ab206461, abcam, Mouse(IgM); 1:200), anti- γH2AX antibody (2577, CST, rabbit; 1:200), anti-P21 antibody (ab109520, Abcam, rabbit; 1:200), anti-eMYHC antibody (Developmental Studies Hybridoma Bank, mouse; 1:500). Immunofluorescence signals were visualized with Alexa488-or Alexa594-conjugated secondary antibodies, using a fluorescence microscope (AXIO observer Z1, Zeiss) equipped with 63×, 40×, and 20× objective lenses. Fluorescence intensity was quantified using ImageJ software for further statistical analyses. For detection of EdU in immunostained samples, the click chemistry reaction was performed after staining with primary and secondary antibodies using a Click-iT EdU Imaging Kit (Life Technologies) according to the manufacturer's instructions.

### 4.9 Myoblast fusion index and measurement of myotube length

Myotubes were identified by positive MF-20 staining. The fusion index was calculated by dividing the number of nuclei incorporated in myotubes by the total number of nuclei observed in each images. Statistical assessment is based on 60 randomly chosen images for each group.

Myotube length was measured using ImageJ. Five representative measurements of myotube length were collected per viewing field. 50 randomly chosen viewing fields were analyzed for each group. For comparisons, the weights of dissected muscles from individual mice were normalized to the respective body weights.

### 4.10 Calcium measurements

Changes in intracellular Ca^2+^ levels were determined by the fluorescent Ca^2+^ -sensitive dye Fluo-4 AM (F14201, Invitrogen), following published protocols [66]. MuSCs were incubated for 30 min s at 37 °C with 5 μM Fluo-4 AM in Ca^2+^ and Mg^2+^free HBSS-HEPES buffer, after which cells were washed 3 times with HBSS-HEPES buffer. Time-lapse images were acquired at every 1.5s with a Leica SP8 MP Microscope using an immersion 40× objective. Excitation wavelength was set to 488 nm, and emission was recorded at 535 ± 15 nm. Analysis and processing of image sequences was done using the ImageJ/FIJI software.

### 4.11 Western blot assay

Freshly isolated or cultured cells were washed with ice-cold PBS and lysed in cell lysis buffer (20 mM Tris (pH 7.5), 400 mM NaCl, 1 mM EDTA, 1 mM EGTA, 1% Triton X-100, 1× Complete Protease Inhibitor Cocktail (Roche Diagnostics)) for 10 min on ice, followed by sonication with Bioruptor (Dianagene) at 4 °C for 5 min. Proteins were separated by SDS-PAGE and transferred to nitrocellulose membranes (Millipore, Billerica, MA). Membranes were probed with the following primary antibodies: anti-P53 antibody (ab26, Abcam, mouse; 1:500), anti-CaV3.1 (PA5-77311, Thermo, rabbit 1: 200), anti-CaV3.2 (PA5-77313, Thermo, rabbit 1: 200), anti-NOX4 (NB110, NOVUS, rabbit 1: 500), and anti-GAPDH antibody (2118, CST, rabbit; 1:200) at 4 °C overnight. Proteins detected by primary antibodies were visualized with HRP-conjugated secondary antibodies using the ECL detection system (Pierce) and quantified using ImageJ software [67].

### 4.12 SA-β-galactosidase detection assay

SA-β-Galactosidase staining was performed as described previously [56]. Cells and sections were fixed for 4 min at RT in a solution of PBS, 1% PFA, 0.2% glutaraldehyde. Samples were washed twice in PBS at pH 7 for 10 min and incubated for 30 min in PBS (pH 6), followed by incubation in X-gal staining solution (4 mM K3Fe(CN)6, 4 mM K4Fe(CN)6, 2 mM MgCl_2_, 0.02% NP-40 and 400 μg/ mL X-gal (15520-018, Sigma) in PBS pH6) at 37 °C. Staining was allowed to proceed overnight for cultured cells and for 48h with a change of the staining solution when sections were used. Samples were washed in PBS, and post-fixed in 1% PFA 5 min for cells and 30 min for sections. After 3 times washing for 10 min with PBS, samples were mounted in PBS, 20% glycerol or processed for immunochemistry.

### 4.13 RNA sequencing

RNAseq was performed as previously described [64]. Samples were generated in biological triplicate and used for RNaseq. Total RNA was isolated using commercial kits and according to the manufacturer's protocols (RNAeasy Mini kit, QIAGEN). RNA integrity of RNA and library preparations were assessed using the LabChip Gx Touch 24 (PerkinElmer). 300 ng of total RNA was used as input for VAHTS Stranded mRNA-seq. Library preparations were done following manufacture's protocol (Vazyme). Sequencing was performed on a NextSeq500 instrument (Illumina) using v2 chemistry, resulting in an average of 30 M reads per library with 1x75bp single end reads. Raw reads were assessed for quality, adaptor content and duplication rates with FastQC 0.10.1, trimmed by Reaper version 13–100 69 and terminally aligned to the Ensemble mouse genome version mm10 (GRCm38) by STAR 2.4.0a. The number of reads aligning to genes was counted with the featureCounts 1.4.5-p1 tool from the Subread package. Only reads mapping at least partially inside exons were admitted and aggregated per gene. Reads overlapping multiple genes or aligning to multiple regions were excluded. Differentially expressed genes were identified using DESeq2 version 1.62 l. Only genes with an absolute fold change of ≥2, a Benjamini-Hochberg corrected *p*-value ≤ 0.05, and a minimum combined mean of 5 reads were assumed to be significantly differentially expressed. Ingenuity Pathway Analysis was performed using QIAGEN's Ingenuity Pathway Analysis algorithm (www.qiagen.com/ingenuity, QIAGEN, Redwood City, CA, USA) for genes with minimum fold changes of ±2 and a Benjamini-Hochberg corrected *p*-value ≤ 0.05. Gene set enrichment analysis was performed to characterize differentially expressed genes (DEGs). Gene sets were extracted from MSigDB. Heatmaps of enriched leading-edge gene clusters were generated using the ‘pheatmap’ and ‘circheatmap’ packages of R. Volcano plots of DEGs between *Piezo1*^*scKO*^ MuSCs and controls were generated using the ‘ggplot2’ package of R. Gene expression data were deposited in the NCBI GEO database under GSE190933 (https://www.ncbi.nlm.nih.gov/geo/query/acc.cgi?acc=GSE190933).

### 4.14 RT-qPCR assays

RT-qPCR analysis were performed using TaqMan gene expression or SYBR Green-based RT-qPCR assays. Total RNA of samples was isolated using commercial kits, following the manufacturer's protocols (RNAeasy Mini kit, QIAGEN). cDNA was generated with the PrimeScript™ II 1st strand cDNA Synthesis Kit (Takara). TaqMan probe-based RT-PCR was performed with the TaqMan Fast Advanced Master Mix (Thermo Fisher). Sequences of TaqMan probes for detection of *Piezo1, MyoD1, Pax7, Calcr* genes are listed in Supplementary Table 1. SYBR Green-based RT-qPCR was performed with the Applied Biosystems™ PowerUp™ SYBR™ Green Master Mix (Thermo Fisher). Sequences of primers to detect expression of *Cacna1g, Cacna1h, Nox1, Nox2, Nox3, Nox4* are listed in Supplementary Table 1. Relative expression levels of genes of interest were determined using the comparative threshold method, using *Gapdh* as internal control. Data were analyzed using the ΔΔCt method [64].

### 4.15 Histological analysis

The muscles were dissected at different time points as indicated in each figure, and snap frozen in isopentane cooled with liquid nitrogen. Sections (8 μm) of muscle samples were prepared with a cryomicrotome and processed for Hematoxylin & Eosin staining as described previously [6].

### 4.16 Statistics

All experiments were performed in biological triplicates unless stated otherwise. Sample sizes for studies was chosen based on observed effect sizes and standard errors. For statistical analysis we either used one-way ANOVA when multiple groups were compared or unpaired two-tailed Student's t-test when two groups were compared. Student's t-test was only used when the variances of the two groups did not differ by factor >3. *P* values < 0.05 were considered statistically significant (∗*p* < 0.05, ∗∗*p* < 0.01). Values are depicted as ± s.e.m. GraphPad Prism 9 was used for data analysis. Calcium transients were calculated as dF/F = (Ft-F)/F, where F is baseline fluorescence intensity and Ft is the intensity of the fluorescence detected in *Piezo1*^*scKO*^ or *Piezo1*^*scKO*^ MuSC. dF/F of 11 ROI (regions of interest) were calculated for each image.

## Declaration of interests

The authors declare that they have no known competing financial interests or personal relationships that could have appeared to influence the work reported in this paper.

## Author contribution

YP, AS and TB conceived and designed experiments. YP and JD performed most of the experiments, analyzed the data, and prepared figures. SG performed next generation RNA sequencing. YD performed bio-informatics analysis. SW provided transgenic mouse lines and advice. LZ supervised animal experiments, TB, AS, YD and JD wrote the manuscript.
