# Supplementary material for: Mechano-signaling via Piezo1 prevents activation and p53-mediated senescence of muscle stem cells
Source: Redox Biol. 2022 Apr 2;52:102309. doi: 10.1016/j.redox.2022.102309 (PMC9005960; doi:10.1016/j.redox.2022.102309)
Supplement: Multimedia component 1 [file mmc1.pdf]

Supplementary Material

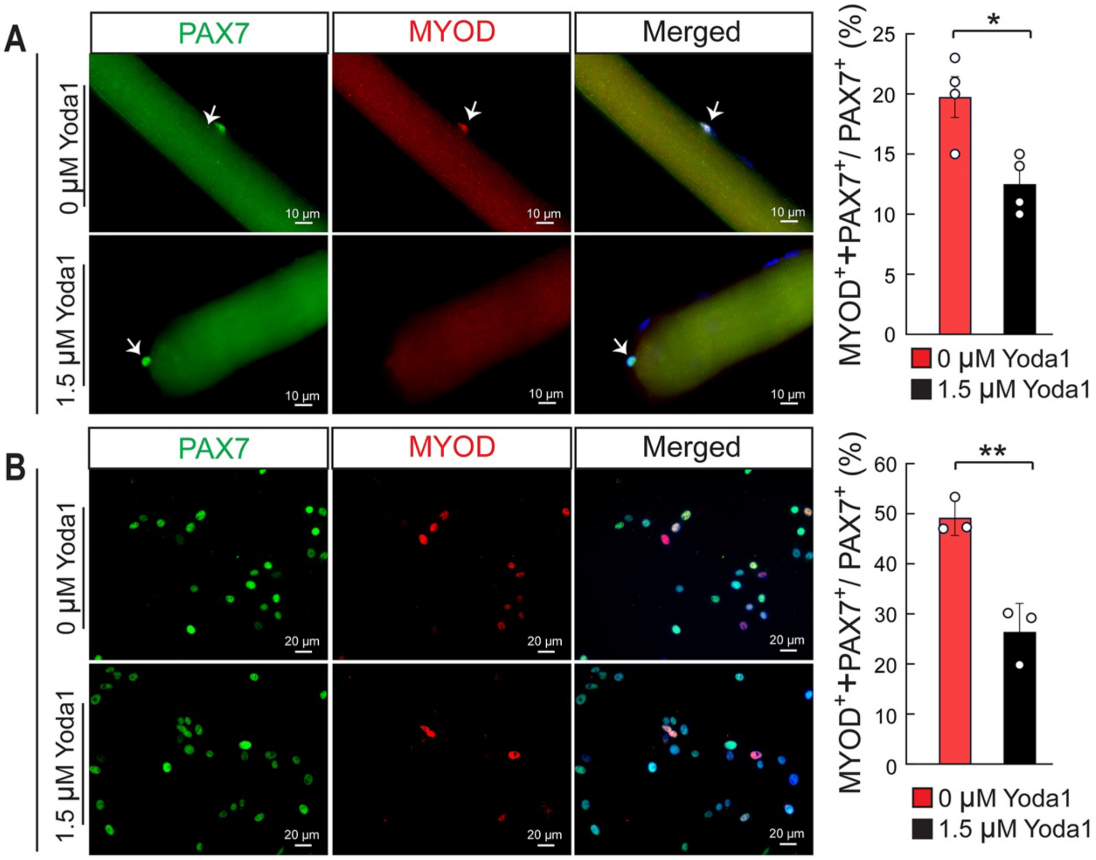

Figure S1. Treatment with the Piezo1 activator Yoda1 prevents activation of MuSCs *in vitro*. (A) Immunofluorescence staining for MYOD (red), PAX7 (green), and DAPI (blue) on isolated FDB myofibers of WT mice treated with 0 μM or 1.5 μM Yoda1 after 8h of culture. Quantification is shown on the right (t-test: **p*<0.05, n=4 independent experiments, ≥100 randomly chosen myofibers per experiment). **(B)** Immunofluorescence staining for MYOD (red), PAX7 (green), and DAPI (blue) of cultured WT MuSCs treated with 0 μM or 1.5 μM Yoda1 for 48h. Quantification is shown on the right (t-test: ***p*<0.01, n=3 independent experiments, 5 technical replicates, 10 fields for each replicate).

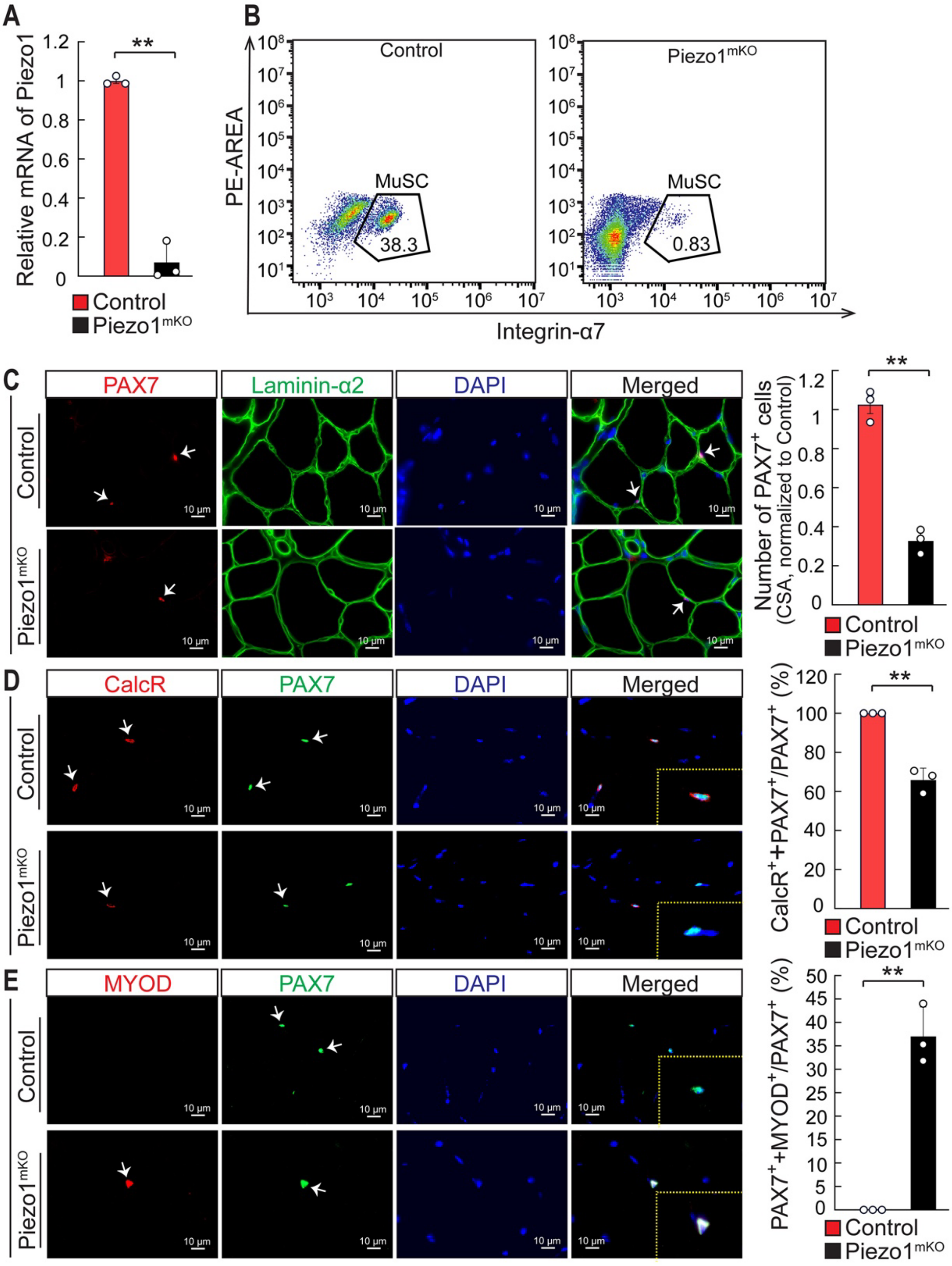

Figure S2. Loss of *Piezo1* leads to activation of MuSCs and diminishes the MuSC pool. (A) RT-qPCR analysis of *Piezo1* expression in isolated control and *Piezo1^{mKO}* MuSCs. *Piezo1* levels in *Piezo1^{mKO}* MuSCs were related to the control group after normalization to *Gapdh* (t-test: ***p*<0.01, n=3, 2-months-old male mice). **(B)** FACS analysis of MuSCs in control and *Piezo1^{mKO}* mice. **(C)** Immunofluorescence staining for PAX7 (red), laminin-α2 (green) and DAPI (blue) in control and *Piezo1^{mKO}* TA muscles. Quantification of PAX7^{+} MuSCs (normalized to controls) is shown on the right (t-test: ***p*<0.01, n=3, 2-months-old male mice, 3 sections per muscle sample). **(D)** Immunofluorescence staining for CALCR (red) PAX7 (green) and DAPI (blue) in control and *Piezo1^{mKO}* TA muscles.

Quantification of $CALR^+$ MuSCs (normalized to controls) is shown on the right (t-test: $^{**}p<0.01$; ns, $p>0.05$, n=3, 2-months-old male mice, 3 sections were quantified for each muscle sample). **(E)** Immunofluorescence staining for MYOD (red) PAX7 (green) and DAPI (blue) control and *Piezo1*^{mKO} TA muscles. Quantification of $MYOD^+$ MuSCs (normalized to controls) is shown on the right (t-test: $^{**}p<0.01$; ns, $p>0.05$, n=3, 2-months-old male mice, 3 sections per muscle sample).

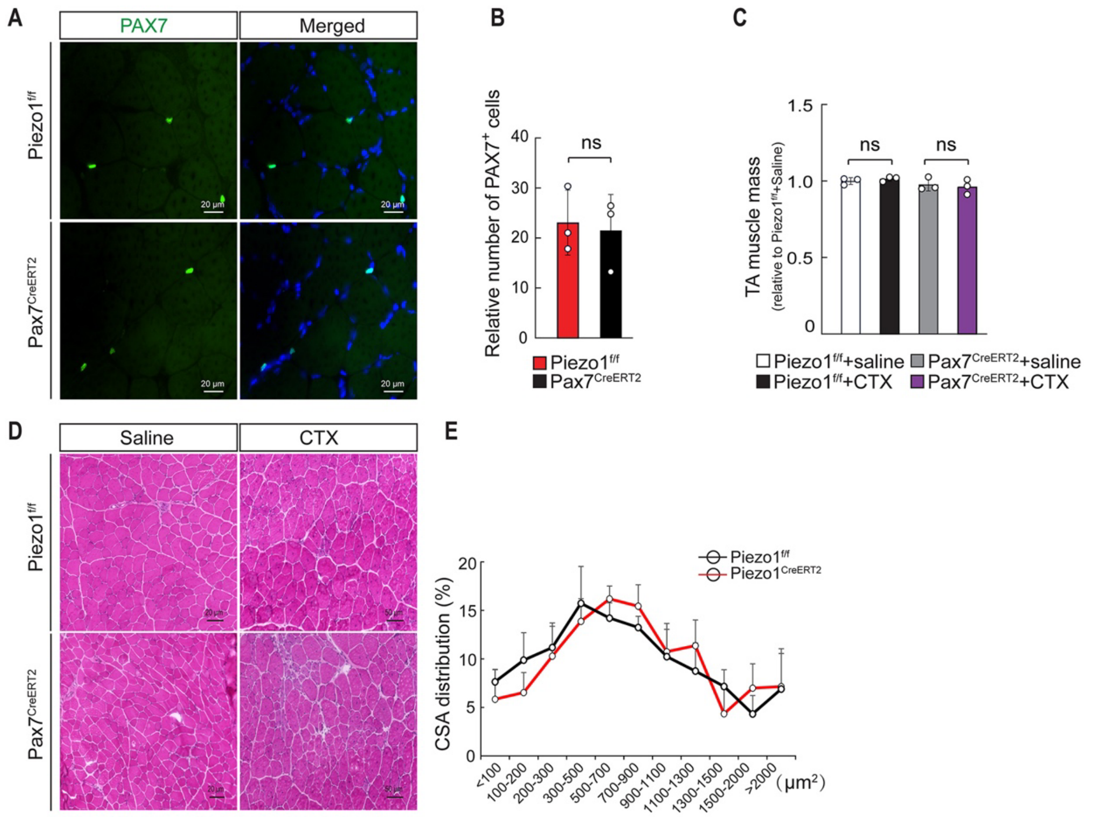

Figure S3. *Piezo1* $^{f/f}$ and *Pax7* ^{CreERT2} mouse have no significant difference in the number of Pax7^{+} cells and muscle regeneration capability. (A) Immunofluorescence staining for PAX7^{+} MuSCs on TA muscle sections from *Piezo1* $^{f/f}$ and *Pax7* ^{CreERT2} mice. **(B)** Quantifications of PAX7^{+} MuSCs on TA muscle sections from *Piezo1* $^{f/f}$ and *Pax7* ^{CreERT2} mice (t-test: ns, p>0.05, n=3, 2-months-old male mice, 3 sections per muscle sample). **(C)** Weights of *Piezo1* $^{f/f}$ and *Pax7* ^{CreERT2} mice TA muscles after CTX-induced muscle injuries at day 14. All values are relative to saline-injected *Piezo1* $^{f/f}$ mice., TA muscle weights were normalized to body weights (One-way ANOVA-test: ns, p>0.05, n=3, 2-months-old male mice, 3 sections per muscle sample). **(D)** H&E staining of non-injured and cardiotoxin-injected TA muscles from of *Piezo1* $^{f/f}$ and *Pax7* ^{CreERT2} TA muscles after CTX-induced muscle injuries at day 14. **(E)** Distribution of myofibers with different cross-sectional areas (CSA) in TA muscles of *Piezo1* $^{f/f}$ and *Pax7* ^{CreERT2} mice 14 days after injury (n=3, 2-months-old male mice).

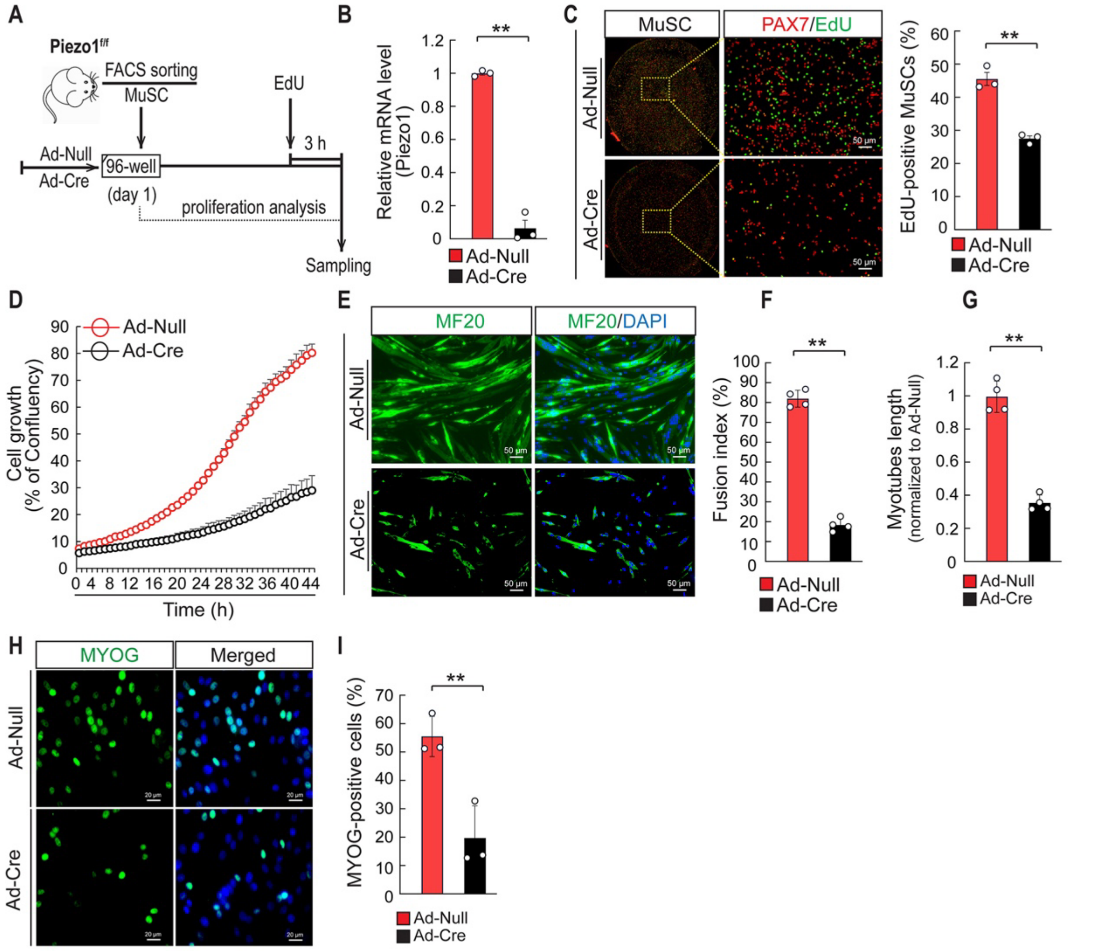

Figure S4. Loss of *Piezo1* attenuates proliferation and differentiation of MuSCs. (A) Schematic outline of Ad-Cre induced *Piezo1* depletion in MuSC. **(B)** RT-qPCR analysis of *Piezo1* expression after Ad-Cre virus infection. Expression levels were normalized to *Gapdh*. (t-test: ***p*<0.01, n=3, 2-months-old male mice) **(C)** Analysis of EdU incorporation in MuSCs after Ad-Cre and Ad-null(up) virus infection of *Piezo1*$^{f/f}$ MuSCs. Quantification of EdU^{+} (red) PAX7^{+} (green) MuSCs at day 5 of culture (t-test: ***p*<0.01, n=3 independent experiments, 4 technical replicates per experiment). **(D)** Time lapse imaging of MuSC proliferation after Ad-Cre and Ad-null infection of *Piezo1*$^{f/f}$ MuSCs. **(E)** Immunofluorescence staining for myosin heavy chain (green, MF20 antibody) and DAPI (blue) at day 5 after induction of differentiation, following Ad-Cre and Ad-null infection of *Piezo1*$^{f/f}$ MuSCs. **(F, G)** Quantifications of fusion index and myotube length of both groups shown in (E) are on the right (t-test: ***p*<0.01, n=4 independent experiments, 10 technical replicates per experiment). **(H, I)** Immunofluorescence staining for MyoG (green) and DAPI (blue) at day 5 after induction of differentiation, following Ad-Cre and Ad-null infection of *Piezo1*$^{f/f}$ MuSCs (H) and quantification (I) (t-test: ***p*<0.01, n=3 independent experiments, 4 technical replicates per experiment).

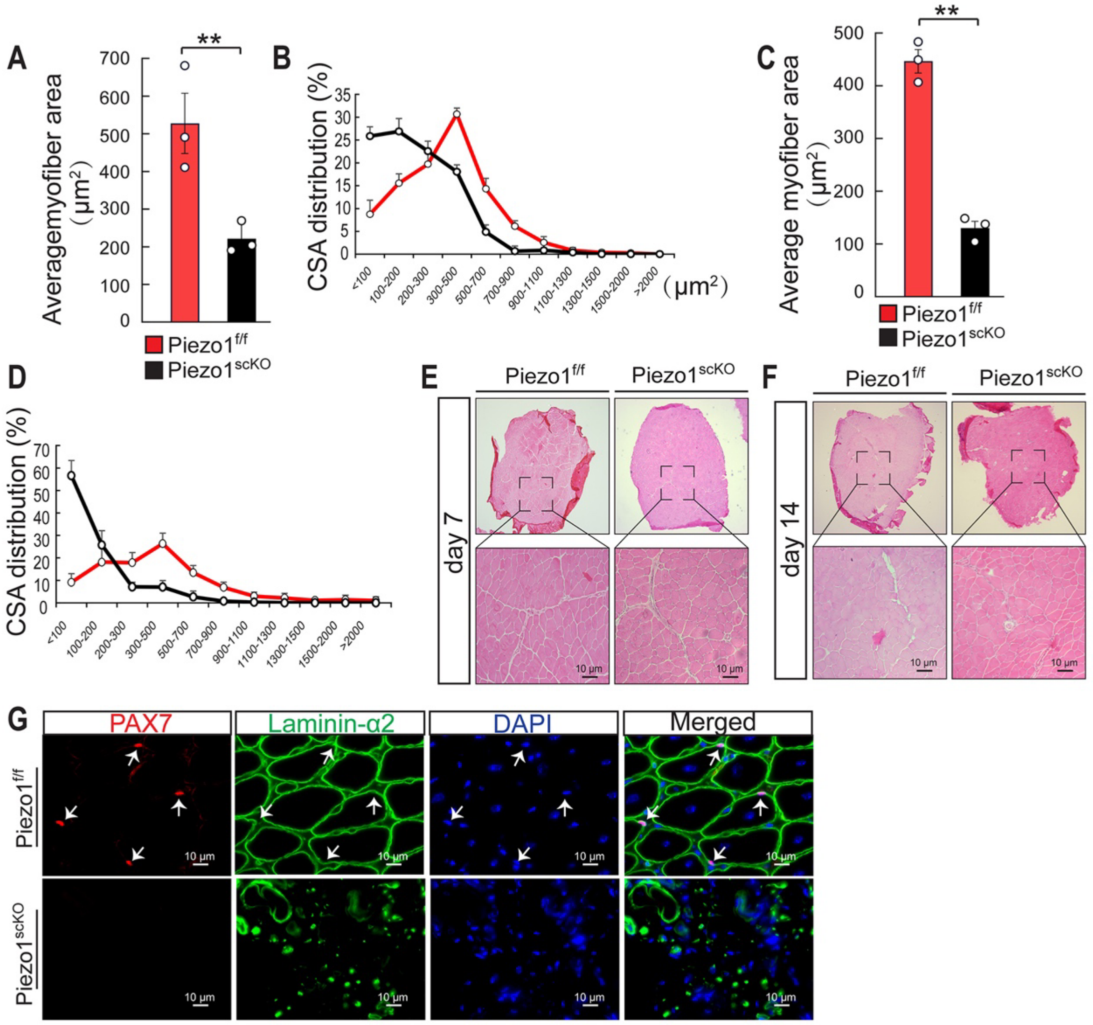

Figure S5. Inactivation of *Piezo1* depletes the MuSC pool after repeated muscle injury (A) Average myofiber area in TA muscles of *Piezo1$^{f/f}$* and *Piezo1^{scKO}* mice 7 days after 1-time CTX injection (t-test: ***p*<0.01, n=3, 2-months-old male mice, 3 sections were quantified for each muscle sample). **(B)** Distribution of myofibers with different cross-sectional areas (CSA) in TA muscles of *Piezo1$^{f/f}$* and *Piezo1^{scKO}* mice after 1-times CTX at day 7. **(C)** Average myofiber area in TA muscles of *Piezo1$^{f/f}$* and *Piezo1^{scKO}* mice after 3-times CTX injections at day 43 (t-test: ***p*<0.01, n=3, 2-months-old male mice, 3 sections were quantified per sample). **(D)** Distribution of myofibers with different cross-sectional areas (CSA) in TA muscles of *Piezo1$^{f/f}$* and *Piezo1^{scKO}* mice after 3-times CTX at day 43. **(E)** H&E staining of muscle sections from non-injured TA muscles of *Piezo1$^{f/f}$* and *Piezo1^{scKO}* mice 7 days after CTX injection. **(F)** H&E staining of muscle sections from non-injured TA muscles of *Piezo1$^{f/f}$* and *Piezo1^{scKO}* mice 14 days after CTX injection. **(G)** Immunofluorescence staining for PAX7 (red), laminin-α2 (green) and DAPI (blue) in *Piezo1$^{f/f}$* and *Piezo1^{mKO}* TA muscles after 3-times CTX injections at day 43.

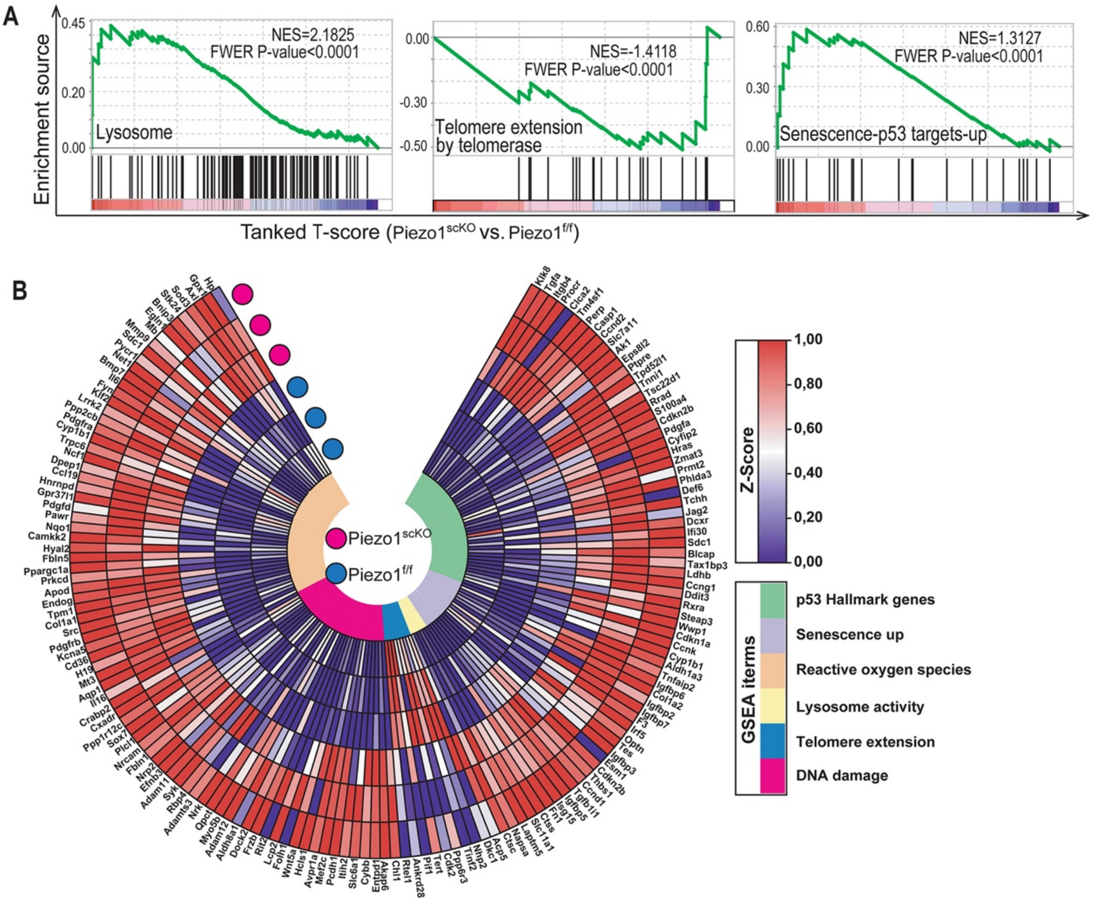

Figure S6. *Piezo1* prevents expression of genes in MuSCs characteristic for cellular senescence. (A) GSEA for lysosome activity, telomere extension and P53 senescence targets based on RNAseq of *Piezo1*$^{f/f}$ and *Piezo1*^{scKO} MuSC. **(B)** Heatmaps of GESA leading edge gene clusters.

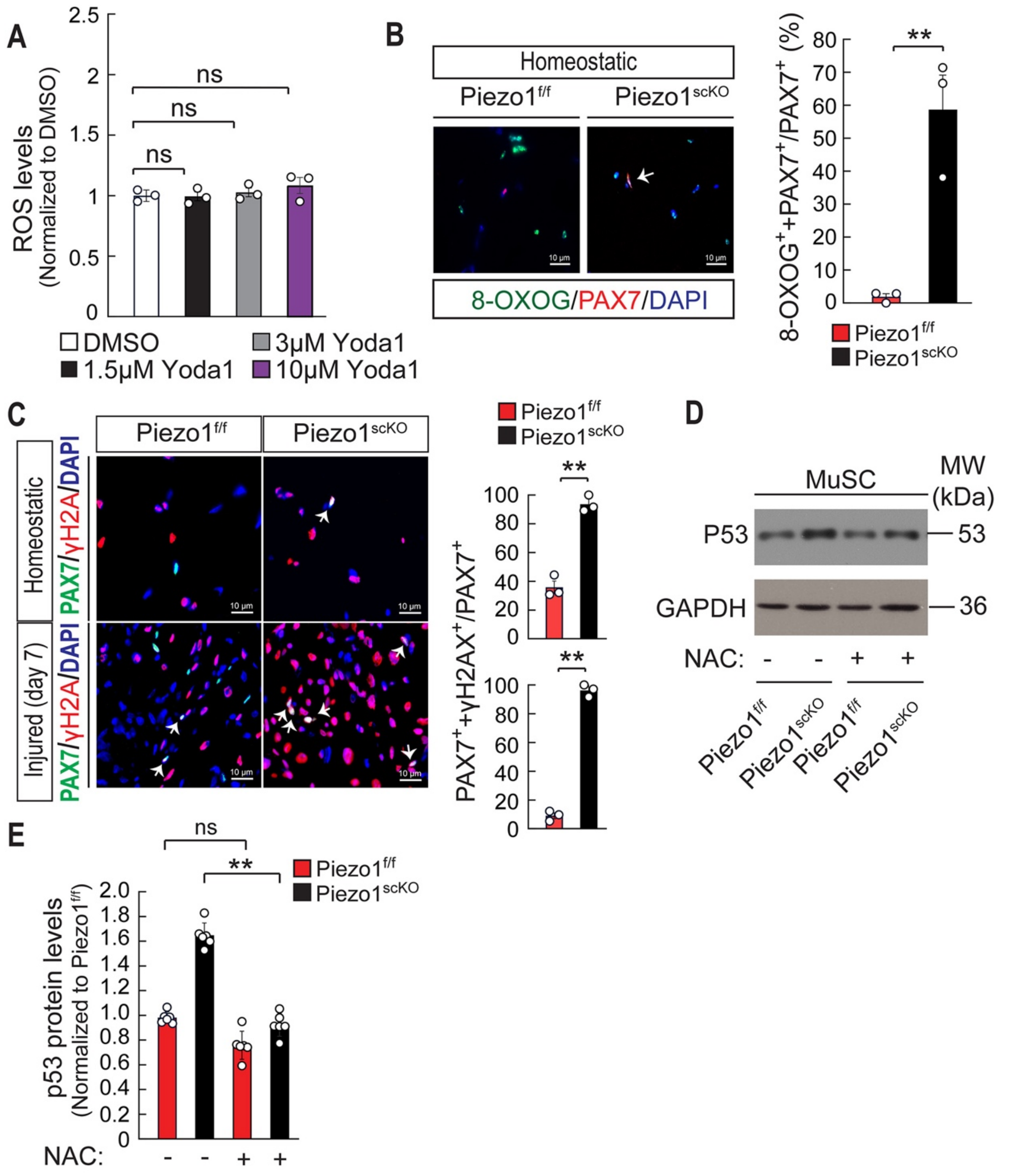

Figure S7. Loss of Piezo1 increases DNA damage in MuSCs. (A) Intracellular ROS concentration in wildtype MuSCs after treatment with 1.5, 3 or 10μM Yoda1 and DMSO for 48h (One-way ANOVA-test: ns, *p*>0.05, n=3 independent experiments, 6 replicates per experiment). **(B)** Detection of 8-oxoguanine (green) in PAX7^{+} MuSCs (red) of *Piezo1$^{f/f}$* and *Piezo1^{scKO}* TA muscles during homeostasis. Nuclei are stained with DAPI (blue). Quantification is shown on the right (t-test: **p*<0.05; ***p*<0.01; ****p*<0.001; ns, *p*>0.05, n=3, 2-months-old male mice, 5 sections were quantified for each muscle sample). **(C)** Immunofluorescence staining for PAX7 (green), γH2AX (red), and DAPI (blue) in *Piezo1$^{f/f}$* and *Piezo1^{scKO}* TA muscles during homeostasis and 7 days after muscle injury. Quantifications are shown on the right (t-test: ***p*<0.01, n=3, 2-months-old male mice, 5 sections were quantified for each muscle sample). **(D; E)** Western blot analysis of P53 levels in *Piezo1$^{f/f}$* and *Piezo1^{scKO}* MuSCs after treatment with 5mM NAC for 24h (D) and quantification (E) (t-test: ***p*<0.01; ns, *p*>0.05, n=6 mice).

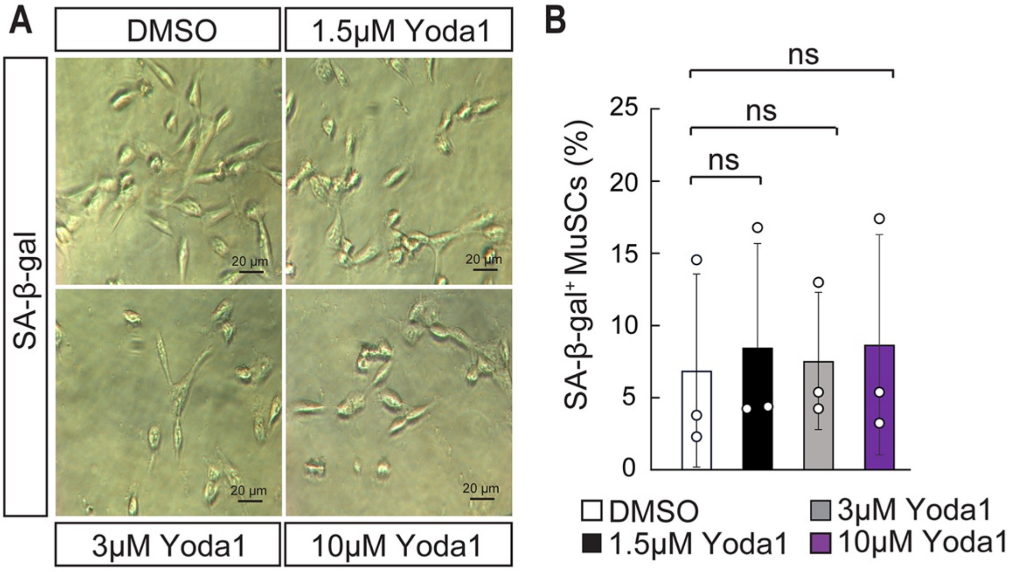

Figure S8. Stimulation of Piezo1 activity by Yoda1 has no effects on senescence of MuSCs. (A) SA-β-gal staining of wild type MuSCs after treatment with 1.5, 3, or 10μM Yoda1 and DMSO for 48h. **(B)** Quantifications of SA-β-gal^{+} MuSCs from wildtype MuSCs after treatment with 1.5, 3, or 10μM Yoda1 and DMSO for 48h. (One-way ANOVA-test: ns, $p>0.05$, n=3 independent experiments, 6 replicates per experiment).

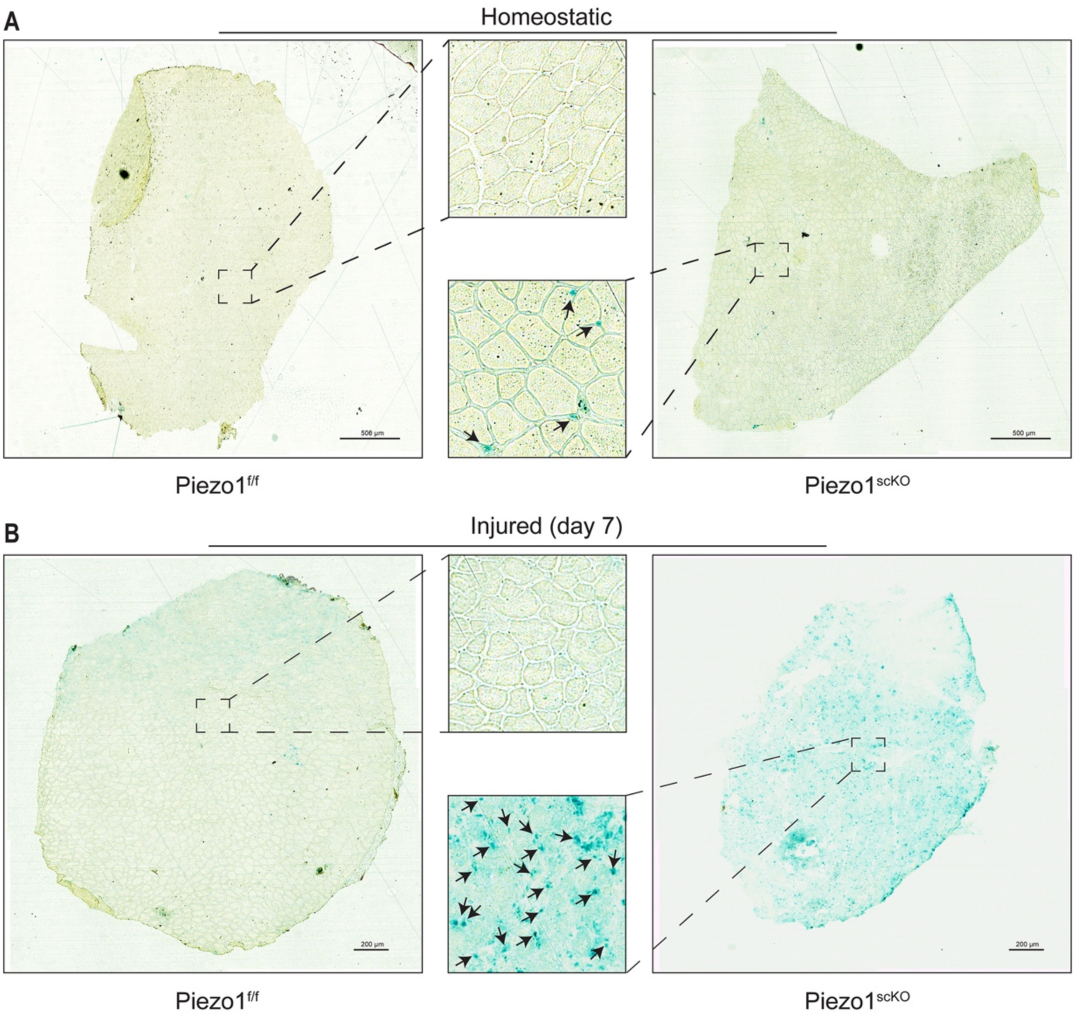

Figure S9. Loss of *Piezo1* increases the number of senescent MuSCs during homeostasis and 7 days after skeletal muscle injury. (A) Low magnification images of SA-β-gal-stained sections from tibialis anterior muscles of *Piezo1*$^{f/f}$ and *Piezo1*^{scKO} mice during homeostasis and **(B)** 7 days after CTX-induced skeletal muscle injury. Arrows indicate examples of SA-β-gal^{+} cells.

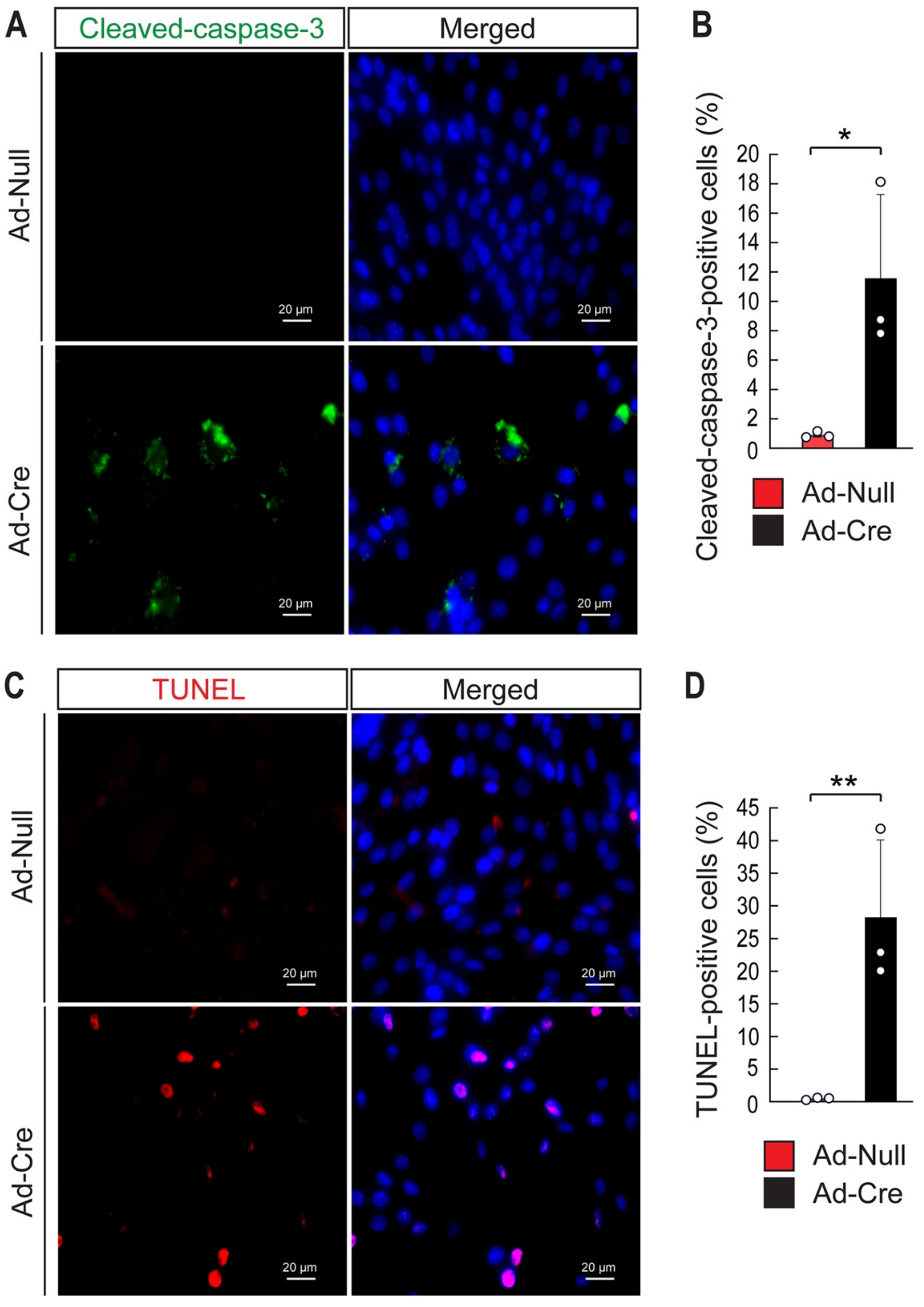

Figure S10. Piezo1 prevents apoptosis of MuSC. (A, B) Immunofluorescence staining for cleaved-caspase-3 (green) and DAPI (blue) 6 days after Ad-Cre and Ad-null infection of *Piezo1*$^{f/f}$ MuSCs (A) and quantification (B) (t-test: *p<0.05, n=3 independent experiments, 6 replicates per experiment). **(C, D)** TUNEL staining (red) and DAPI (blue) 6 days after Ad-Cre and Ad-null infection of *Piezo1*$^{f/f}$ MuSCs (C) and quantification (D) (t-test: **p<0.01, n=3 independent experiments, 6 replicates for each experiment).

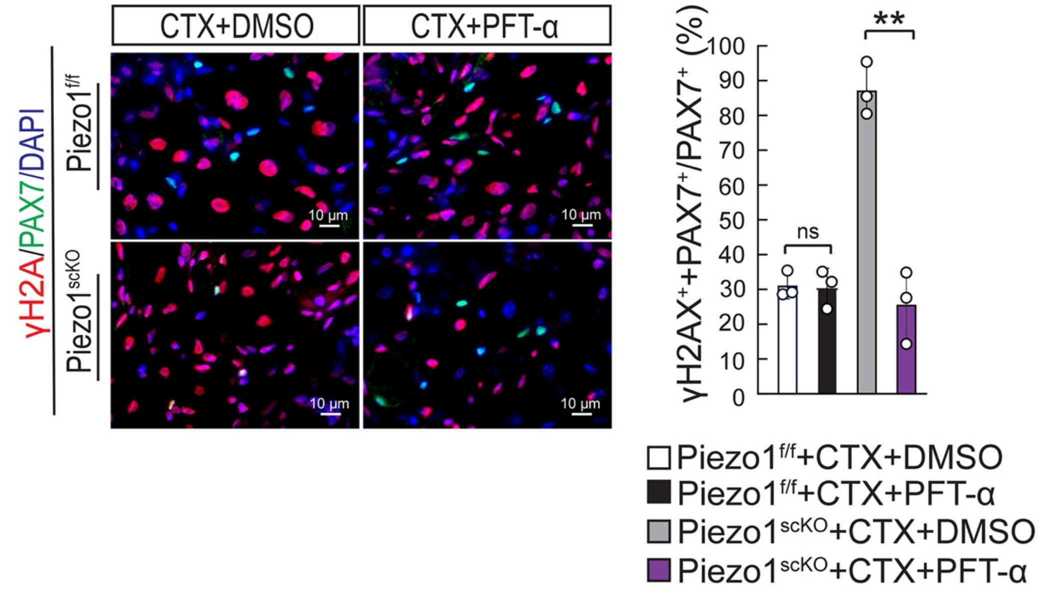

Figure S11. Inhibition of p53 reduces the number of γH2AX⁺ MuSCs in *Piezo1^{scKO}* mice. Immunofluorescence staining for γH2AX (red), PAX7 (green), and DAPI (blue) in *Piezo1$^{f/f}$* and *Piezo1^{scKO}* TA muscles 7 days after CTX-induced skeletal muscle injury, without and with PFT-α treatment. Quantifications are shown on the right (t-test: ***p*<0.01, n=3, 2-months-old male mice, 3 sections were quantified for each muscle sample).

Supplementary table 1. List of primers or Taqman probes used in this paper

Gene/ Primer name	Species	Sequence/ Probe ID	Usage
Pax7	Mouse	mm01354484_m1, Thermo Fisher	Taqman assay
MyoD1	Mouse	Mm00440387_m1, Thermo Fisher	Taqman assay
Calcr	Mouse	mm00432282_m1, Thermo Fisher	Taqman assay
Piezo1	Mouse	Mm01241549_m1, Thermo Fisher	Taqman assay
Gapdh	Mouse	Mm999999915_g1, Thermo Fisher	Taqman assay
Cacna1g	Mouse	Forward: AGATCCCTCTAGCTGAGATGGA<br>Reverse: GGGCACTAAGTAAGAGTGTGTG	RT-qPCR
Cacna1h	Mouse	Forward: CGTGACACTGGGCATGTTC<br>Reverse: CCACCATCTTGATAACCATCTCC	RT-qPCR
Nox1	Mouse	Forward: GGTTGGGGCTGAACATTTTTC<br>Reverse: TCGACACACAGGAATCAGGAT	RT-qPCR
Nox2	Mouse	Forward: TGTGGTTGGGGCTGAATGTC<br>Reverse: CTGAGAAAGGAGAGCAGATTTCG	RT-qPCR
Nox3	Mouse	Forward: CAACGCACAGGCTCAAATGG<br>Reverse: CACTCTCGTTCAGAATCCAGC	RT-qPCR
Nox4	Mouse	Forward: GAAGGGGTTAAACACCTCTGC<br>Reverse: ATGCTCTGCTTAAACACAATCCT	RT-qPCR
Gapdh	Mouse	Forward: AGGTCGGTGTGAACGGATTTG<br>Reverse: TGTAGACCATGTAGTTGAGGTCA	RT-qPCR
***Piezo1* loxp**	Mouse	Forward:<br>CCAGTGATTCCTCATGGAATGTGG<br>Reverse:<br>CTTAAGCCCATCTCACAGCTGAAGC	Genotyping
***Pax7*CreErt2**	Mouse	Forward:<br>GCT GCT GTT GAT TAC CTG GC<br>Reverse1:<br>GCA CTG AGA CAG GAC CG<br>Reverse2:<br>CAA AAG ACG GCA ATA TGGTG	Genotyping
Pax7-Cre	Mouse	Forward:<br>CTG CGT TTC TCC GAG CTG CAG<br>Reverse:<br>CCT CGT CGT CCT CTT TCT TCCC	Genotyping
